# Circadian–melatonin signaling in general anesthesia: molecular mechanisms and perioperative implications

**DOI:** 10.3389/fphar.2026.1960152

**Published:** 2026-10-15

**Authors:** Wui-Chiu Mui, Yi-Chou Hou, Yi-Cheng Liao, Kuo-Cheng Lu

**Affiliations:** 1 Department of Anesthesiology, Ditmanson Medical Foundation Chia-Yi Christian Hospital, Chiayi, Taiwan; 2 School of Medicine, College of Medicine, Fu Jen Catholic University, New Taipei City, Taiwan; 3 Division of Nephrology, Department of Internal Medicine, Cardinal-Tien Hospital, School of Medicine, College of Medicine, Fu Jen Catholic University, New Taipei City, Taiwan; 4 Division of Nephrology, Department of Medicine, Taipei Tzu Chi Hospital, Buddhist Tzu Chi Medical Foundation, New Taipei City, Taiwan; 5 Division of Nephrology, Department of Internal Medicine, Fu Jen Catholic University Hospital, New Taipei City, Taiwan

**Keywords:** circadian rhythm, general anesthesia, melatonin, molecular clock, perioperative medicine, postoperative delirium, ramelteon

## Abstract

Anesthesia and surgery are delivered to a temporally organized organism whose physiology is governed by the suprachiasmatic nucleus, peripheral molecular clocks, and pineal melatonin signaling. This review synthesizes mechanistic, preclinical, and clinical evidence on how anesthetic agents, surgical stress, and the perioperative environment interact with circadian and melatonergic signaling, and on whether that interaction can be targeted therapeutically. In rodent and cellular studies, volatile and intravenous anesthetics alter clock function in an agent- and phase-dependent manner; proposed mechanisms include GABAergic and glutamatergic modulation of entrainment, transcriptional and chromatin-level repression of CLOCK–BMAL1/PER–CRY signaling, and metabolic coupling through CaMK–CREB, AMPK, SIRT1, and GSK-3β. These mechanisms have not been confirmed in humans. In patients, these effects are inseparable from surgical injury, opioids, fasting, inflammation, and disrupted light–dark exposure, all of which suppress melatonin amplitude, delay its onset, and promote internal desynchrony. Melatonin signals through MT_1_ and MT_2_ receptors to produce chronobiotic, anxiolytic, and antinociceptive effects, whereas the mitochondrial, redox, and anti-inflammatory actions, described almost exclusively in animal and cellular models, require substantially higher exposures and have not been demonstrated in surgical patients; the two arms are pharmacologically distinct and should not be pooled. Clinically, preoperative anxiolysis with melatonin in adults is the most consistently supported indication, whereas evidence for postoperative sleep, analgesia, delirium prevention, and organ protection remains heterogeneous and of low certainty. Most trials have used fixed clock-time dosing without measuring internal circadian phase or achieved plasma exposure, so null results cannot distinguish an ineffective mechanism from an inadequate intervention. The circadian–melatonin axis remains a plausible perioperative target, but clinical translation requires phase-aware, exposure-informed, and adequately powered trials.

## 1 Introduction

The circadian system coordinates sleep–wake timing, neuroendocrine and metabolic activity, immune signaling, and tissue repair, and recent reviews indicate that anesthesia and surgery disturb this temporal organization (Guo et al., 2023; Patel and Ownby, 2025). Melatonin serves both as an endocrine marker of biological night and as a candidate therapeutic signal, and dim-light melatonin onset remains the reference method for assessing human circadian phase (Murray et al., 2024). The perioperative period concentrates multiple circadian stressors—fasting, pain, opioids, altered light exposure, sleep fragmentation, immobility, and intensive-care interventions—that can suppress rhythm amplitude, disturb sleep–wake organization, and desynchronize central and peripheral clocks (Patel and Ownby, 2025; Wilcox et al., 2024). Time-of-day differences in surgical outcome have been reported, but the evidence is of low certainty and does not support routine clinical chronoscheduling (Meewisse et al., 2024).

Preclinical and translational studies indicate that general anesthetics disturb circadian organization through agent- and time-of-day-dependent mechanisms (Guo et al., 2023; Sugimura et al., 2024). A comparative murine study found that sevoflurane, desflurane, and isoflurane produce distinct changes in locomotor circadian rhythms according to agent and timing of exposure (Sugimura et al., 2024). Two mechanisms have been characterized in detail in rodents—sevoflurane-associated loss of CLOCK occupancy and histone H4 acetylation at the *Per2* promoter (Mori et al., 2014) and propofol-associated changes in CaMK–CREB, AMPK, SIRT1, and GSK-3β signaling (Yin et al., 2022)—although both derive from single research groups and neither has been independently replicated or examined in humans.

Melatonin has consequently been examined both as a perioperative circadian biomarker and as a therapeutic adjunct. Its MT_1_/MT_2_ receptor-mediated chronobiotic actions should be distinguished from the mitochondrial, mitophagic, and redox effects described experimentally, which may require substantially higher exposures (Woldanska-Okonska and Koszela, 2024; Rahmani et al., 2024). The clinical evidence is heterogeneous: meta-analyses suggest that melatonin, but not ramelteon, may reduce postoperative delirium in selected surgical populations, whereas several recent randomized trials and intensive-care studies report inconsistent or null results (Barnes et al., 2023; Borges et al., 2026; Tang et al., 2025; Mekontso Dessap et al., 2025; Khaled et al., 2025; Elbakry et al., 2024; Shin et al., 2024; Jiang et al., 2023), and postoperative sleep benefits depend on the outcome measure, dose, formulation, and timing of administration (Tsukinaga et al., 2023). Preoperative anxiolysis in adults remains the most consistently supported indication (Madsen et al., 2020). Because fixed clock-time dosing does not account for internal circadian phase, chronotype, or formulation-dependent exposure, future trials should combine validated phase assessment with pharmacokinetic, environmental, and clinically relevant endpoints (Chen et al., 2025; Harpsoe et al., 2015). This review examines molecular circadian–melatonin signaling, agent-specific anesthetic effects, perioperative rhythm disruption, and the current clinical evidence, and proposes a phase-aware, exposure-informed framework for perioperative chronomedicine.

### 1.1 Scope and literature search

This is a narrative rather than a systematic review, but literature identification was structured to improve transparency. We searched PubMed/MEDLINE, Embase, the Cochrane Library, and Web of Science from database inception to 30 June 2026 for English-language articles, supplemented by manual screening of the reference lists of key reviews and trials and by forward citation tracking. Search terms combined circadian terms (circadian, clock gene, CLOCK, BMAL1, PER, CRY, suprachiasmatic nucleus, chronotype, dim-light melatonin onset), melatonergic terms (melatonin, ramelteon, MT_1_, MT_2_, MTNR1B), and perioperative terms (anesthesia/anaesthesia, sevoflurane, isoflurane, desflurane, propofol, dexmedetomidine, ketamine, remimazolam, surgery, intensive care, postoperative delirium, perioperative neurocognitive disorders). For clinical questions, we prioritized, in order, (i) systematic reviews and meta-analyses of randomized trials and large or multicenter randomized trials; (ii) smaller randomized trials and prospective studies with objective circadian or melatonin measurements; and (iii) retrospective or cross-sectional studies. For mechanistic questions, we prioritized *in vivo* studies that reported zeitgeber time, anesthetic concentration, and exposure duration, and used cellular and *ex vivo* studies to illustrate mechanism rather than to support clinical inference. When a more recent synthesis superseded an earlier one, the more recent synthesis was given priority; earlier syntheses were retained only when they addressed a different population or question. Conference abstracts, non-peer-reviewed preprints, and reports without accessible full text were excluded. No formal risk-of-bias assessment or quantitative synthesis was performed (Section 11). Table 1 classifies the evidence underlying each principal claim of this review by species, study design, anesthetic exposure, melatonergic agent and formulation, and certainty, so that clinical and preclinical findings can be distinguished at a glance.

**TABLE 1:** Evidence map: level of evidence underlying the principal claims of this review.

Claim or topic	Species and setting	Study design	Anesthetic/perioperative exposure	Melatonergic agent, formulation, dose	Certainty and evidence category	Refs
A. Human randomized trials and meta-analyses						
Preoperative anxiolysis (adults)	Adults undergoing surgery	Cochrane SR/MA; 27 RCTs (n = 2,319)	Various, mostly general anesthesia	Melatonin, oral IR; 3–10 mg or 0.05–0.4 mg/kg	Moderate†; established	(Madsen et al., 2020)
Preoperative anxiolysis (children)	Anxious children; 20 UK centers	Noninferiority RCT, stopped early (n = 110)	General anesthesia	Melatonin 0.5 mg/kg (max 20 mg) vs. midazolam	Low–moderate (imprecision); noninferiority not shown	(Bolt et al., 2024)
POD prevention: melatonin, surgical	Older surgical patients (hip fracture, cardiac, other)	RCTs; pairwise meta-analyses	General and neuraxial anesthesia	Melatonin, oral IR, mostly 3–5 mg for 1–7 days	Low; inconsistent; dose-related signal is a hypothesis	(Borges et al., 2026; Shin et al., 2024; de Jonghe et al., 2014; Ford et al., 2020; Sultan, 2010; Campbell et al., 2019)
POD prevention: ramelteon, surgical	Older surgical patients	RCTs; meta-analysis subgroup	General anesthesia; orthopedic surgery	Ramelteon 8 mg nightly	Low; no consistent benefit (pooled RR 0.98)	(Borges et al., 2026; Kinouchi et al., 2023; Oh et al., 2021)
Delirium prevention: ramelteon, acute medical	Older acutely admitted inpatients (ICU and ward)	Single RCT (n = 67)	None (non-surgical)	Ramelteon 8 mg nightly, up to 7 days	Low (small sample, wide CI); not replicated in surgical trials	(Hatta et al., 2014)
ICU delirium prevention	Critically ill adults, including mechanically ventilated patients	RCTs; SR/MA (24 RCTs)	Sedation, mechanical ventilation	Melatonin or ramelteon, various doses	Low†; modest pooled effect (RR 0.77); DEMEL RCT null	(Tang et al., 2025; Mekontso Dessap et al., 2025; Aiello et al., 2023; Chaves-Filho et al., 2026)
Postoperative sleep	Surgical patients	SR/MA with trial sequential analysis	Various	Melatonin, various regimens	Low; mostly questionnaire outcomes	(Tsukinaga et al., 2023)
Postoperative analgesia	Surgical patients	SR/MA of double-blind RCTs	Various	Melatonin, mostly single preoperative dose	Low; benefit attenuated in higher-quality trials	(Oh et al., 2020; Andersen et al., 2014)
Anesthetic sparing (induction dose)	ASA I adults	Two single-center RCTs	Propofol or thiopental induction	Melatonin 0.2 mg/kg or 3–5 mg oral premedication	Low; not independently replicated	(Naguib et al., 2006; Turkistani et al., 2007)
Sleep architecture with dexmedetomidine	Older ICU patients after non-cardiac surgery; healthy volunteers	RCTs; PK–PD RCT	Low-dose nocturnal dexmedetomidine	None (melatonin measured as outcome)	Moderate for selected sleep outcomes; nocturnal melatonin unchanged	(Akeju et al., 2018; Schnider et al., 2025; Wu et al., 2016; Su et al., 2016; Deiner et al., 2017)
Time of day of cardiac surgery	Cardiac surgery patients	Propensity cohort + RCT substudy; replication cohorts; Cochrane review	On-pump cardiac surgery	Not melatonin (REV-ERBα pathway)	Low; inconsistent replication	(Montaigne et al., 2018; Nemeth et al., 2021; Gotte et al., 2020; Liu et al., 2026)
Short-term safety	Adults	Systematic reviews of RCTs	Various	Melatonin	Low–moderate for short-term tolerability; uncommon harms not assessable	(Madsen et al., 2020; Andersen et al., 2016)
B. Human observational and physiological studies						
Perioperative melatonin suppression and phase delay	Adults; minor and major surgery	Small randomized comparison (n = 20) and prospective cohorts	General vs. spinal anesthesia; surgery	Endogenous melatonin (outcome)	Low; direction consistent, attribution confounded; supported but unconfirmed	(Gogenur et al., 2007a; Gogenur et al., 2007b; Karkela et al., 2002; van et al., 2022)
Anesthetic technique, melatonin rhythm, and POD	Older hip-fracture patients (n = 138)	Prospective non-randomized cohort	General vs. subarachnoid anesthesia	Endogenous melatonin	Low (confounding by indication); not supported by REGAIN RCT	(Song et al., 2021; Neuman et al., 2021)
Loss of melatonin rhythm in ICU	Sedated, ventilated, or septic ICU patients	Observational	Continuous sedation; mechanical ventilation	Endogenous melatonin	Low; supported but unconfirmed	(Mekontso Dessap et al., 2025; Mundigler et al., 2002)
Propofol and rest–activity rhythm	Colonoscopy patients	Observational	Light propofol sedation (low trauma)	Endogenous	Low; single study	(Dispe et al., 2009)
MTNR1B genotype and glycemic response	Population cohorts; normoglycemic women (n = 17)	Genetic association; small experimental study	None (non-surgical)	Melatonin 5 mg	Strong genetic association; perioperative relevance is a hypothesis	(Lyssenko et al., 2009; Tuomi et al., 2016; Garaulet et al., 2015)
C. *In vivo* animal studies						
Sevoflurane: Per2 repression; reduced CLOCK occupancy and H4 acetylation	Mouse, rat	*In vivo*; one research group	2.5% sevoflurane for 1–4 h; time-of-day comparison	None	Preclinical; consistent within one laboratory; not independently replicated	(Mori et al., 2014; Kadota et al., 2012; Ohe et al., 2011; Anzai et al., 2013)
Desflurane and isoflurane: phase shifts and clock-gene changes	Mouse	*In vivo*	Exposure at different times of day	None	Preclinical; phase-dependent	(Sugimura et al., 2024; Gokmen et al., 2017)
Propofol: CaMK–CREB/metabolic signaling; melatonin rescue	Rat	*In vivo*	Prolonged propofol anesthesia	Melatonin pretreatment (rodent weight-adjusted dosing)	Preclinical; rescue shown in a single study	(Yin et al., 2022; Yoshida et al., 2009)
Melatonin and anesthesia-/surgery-associated cognitive impairment; NLRP3	Mouse, including aged mice	*In vivo*	Isoflurane; sevoflurane ± laparotomy	Melatonin, high weight-adjusted doses	Preclinical; relevance to 3–5 mg oral dosing uncertain	(Xia et al., 2016; Yang et al., 2024; Zhu et al., 2023; Jia et al., 2024)
Myocardial and hepatic ischemia–reperfusion protection	Rat, mouse	Isolated heart; *in vivo* ischemia–reperfusion	Ischemia–reperfusion models (not anesthesia-specific)	Melatonin, experimental doses	Preclinical; no confirmation in surgical patients	(Petrosillo et al., 2009; Zhou et al., 2017; Wang et al., 2020; Shi et al., 2024)
D. Cellular and *ex vivo* studies						
Direct sevoflurane action on the SCN	Rat SCN explants	*Ex vivo* bioluminescence	Sevoflurane	None	Mechanistic	(Anzai et al., 2013; Matsuo et al., 2016)
Ketamine and CLOCK–BMAL1 function	Cell lines	*In vitro*	Ketamine	None	Mechanistic; perioperative relevance is a hypothesis	(Bellet et al., 2011)
Receptor-independent redox and mitochondrial actions	Cells; isolated mitochondria	*In vitro*	Not applicable	Melatonin, high concentrations	Mechanistic; exposure-dependent; hypothesis for clinical dosing	(Reiter et al., 2016; Tan et al., 2016)

† Certainty as rated (GRADE) by the source systematic review; all other certainty ratings are the authors’ qualitative judgments, based on design, consistency, precision, and directness. Evidence categories: established, consistent randomized human evidence of at least moderate certainty; supported but unconfirmed, human observational or consistent *in vivo* animal data without randomized confirmation; hypothesis, single model or laboratory, cellular data, or mechanistic inference. ASA, american society of anesthesiologists; CI, confidence interval; ICU, intensive care unit; IR, immediate release; PK–PD, pharmacokinetic–pharmacodynamic; POD, postoperative delirium; RCT, randomized controlled trial; RR, risk ratio; SCN, suprachiasmatic nucleus; SR/MA, systematic review/meta-analysis.

### 1.2 Conceptual framework

Four distinctions organize the remainder of this review. First, general anesthesia is not physiological sleep. Natural sleep and general anesthesia share selected arousal nodes but are not interchangeable states (Franks, 2008; Hemmings et al., 2019). Sleep is cyclic, homeostatically regulated, and organized into non-rapid-eye-movement (NREM) and rapid-eye-movement (REM) stages, whereas anesthetics produce unconsciousness, amnesia, immobility, and antinociception through agent-specific actions on ligand-gated and voltage-gated channels, two-pore-domain potassium channels, synaptic release, and arousal networks, with electroencephalographic signatures that vary by agent, dose, age, and surgical stimulation (Franks, 2008; Hemmings et al., 2019). Several hours of anesthesia therefore do not repay sleep debt and may themselves disturb subsequent sleep architecture (Franks, 2008; Hemmings et al., 2019). Dexmedetomidine, which recruits endogenous NREM-promoting circuitry, is a partial exception at the level of sleep architecture but not demonstrably at the level of circadian phase (Section 4.8) (Akeju et al., 2018; Schnider et al., 2025). Second, sleep and circadian phase are separable. Postoperative sleep quality, the timing and amplitude of the melatonin rhythm, and delirium are related but distinct endpoints; an intervention that improves one need not improve the others, and the circadian consequences of anesthesia are likely to depend on the timing and duration of exposure, surgical injury, temperature, analgesia, and the post-anesthetic environment (Gogenur et al., 2007a; Gogenur et al., 2007b). Third, administered dose is not biological exposure, because oral melatonin bioavailability, formulation, and CYP1A2-dependent clearance vary widely between patients (Section 2.5). Fourth, receptor-mediated chronobiotic, hypnotic, and anxiolytic effects, which occur at low exposures, should be distinguished from the mitochondrial, redox, and anti-inflammatory effects described experimentally at substantially higher exposures (Section 2.4). Throughout, we also identify the melatonergic agent concerned—melatonin or ramelteon—because the two differ in pharmacology and evidence base (Table 2).

**TABLE 2:** Melatonin and ramelteon: comparative pharmacology and perioperative evidence.

Feature	Melatonin	Ramelteon	Refs
Nature and regulatory status	Endogenous indoleamine; sold as a dietary supplement in some countries and as a licensed medicine (e.g., prolonged-release 2 mg) in others; product content varies	Synthetic tricyclic MT_1_/MT_2_ agonist; licensed for sleep-onset insomnia (8 mg)	(U.S. Food and Drug Administration, 2005; Erland and Saxena, 2017)
Receptor profile	MT_1_ and MT_2_ agonist; also binds the MT_3_/QR2 site	Selective MT_1_/MT_2_ agonist with higher affinity than melatonin; low affinity for the MT_3_/QR2 site	(Dubocovich et al., 2010; Jockers et al., 2016; Kato et al., 2005)
Receptor-independent actions	Redox, mitochondrial, and anti-inflammatory actions described experimentally at high exposures	Not shown	(Reiter et al., 2016; Tan et al., 2016)
Oral pharmacokinetics	Bioavailability about 15% (IR), with wide variability; half-life about 45 min (IR); prolonged-release formulations extend exposure	Bioavailability about 2% (extensive first pass); short half-life of the parent drug; active metabolite M-II has a longer half-life and higher systemic exposure	(Lane and Moss, 1985; DeMuro et al., 2000; Burgess et al., 2008)
Metabolism and interactions	Mainly CYP1A2; exposure increased by caffeine and fluvoxamine, reduced by smoking	Mainly CYP1A2 (minor CYP2C and CYP3A4); co-administration with fluvoxamine contraindicated	(Hartter et al., 2003; Burgess et al., 2008; Hartter et al., 2000)
Perioperative regimens studied	IR 3–10 mg or 0.05–0.5 mg/kg preoperatively; 3–5 mg nightly for 1–7 days postoperatively	Mostly 8 mg nightly	(Madsen et al., 2020; de Jonghe et al., 2014; Oh et al., 2021; Hatta et al., 2014)
Preoperative anxiolysis	Adults: moderate-certainty reduction vs. placebo; similar to benzodiazepines. Children: not noninferior to midazolam	Not established; no adequate perioperative trials identified	(Madsen et al., 2020; Bolt et al., 2024)
Postoperative delirium (surgical RCTs)	Inconsistent; largest RCT null; pooled RR 0.67 (0.52–0.88), stronger at ≥5 mg; low certainty	No benefit in RCTs after general anesthesia or orthopedic surgery; pooled RR 0.98 (0.47–2.01)	(Borges et al., 2026; de Jonghe et al., 2014; Ford et al., 2020; Kinouchi et al., 2023; Oh et al., 2021)
Delirium (medical and ICU populations)	Benefit in one earlier trial in medical inpatients but not in a later one; null in the DEMEL ICU trial; no effect on severity of established delirium	Large effect in one small RCT in acutely admitted older adults; shorter ICU stay in one single-center RCT	(Mekontso Dessap et al., 2025; Hatta et al., 2014; Al-Aama et al., 2011; Jaisw et al., 2018; Lange et al., 2024; Nishikimi et al., 2018; Chaves-Filho et al., 2026)
Postoperative sleep, analgesia, anesthetic sparing	Modest or inconsistent effects on sleep and analgesia; reduced induction dose in two small RCTs	Not studied systematically in surgical patients	(Tsukinaga et al., 2023; Oh et al., 2020; Naguib et al., 2006; Turkistani et al., 2007)
Organ protection	Preclinical only	No data	(Petrosillo et al., 2009; Zhou et al., 2017; Wang et al., 2020; Shi et al., 2024)
Interpretive implication	Receptor-mediated and, at high exposures, possibly receptor-independent effects; exposure depends strongly on formulation and CYP1A2 activity	A test of receptor-mediated effects alone; negative ramelteon trials do not rule out effects of high-dose melatonin, and *vice versa*	—

ICU, intensive care unit; IR, immediate release; PI, prescribing information; QR2, quinone reductase 2; RCT, randomized controlled trial; RR, risk ratio. Pooled risk ratios are from Borges et al. (2026).

We follow the consensus nomenclature for cognitive change associated with anesthesia and surgery (Evered et al., 2018): perioperative neurocognitive disorders (PND) is used as the umbrella term; postoperative delirium (POD) denotes delirium occurring up to 1 week after surgery or until discharge; and delayed neurocognitive recovery (dNCR) and postoperative neurocognitive disorder denote cognitive decline diagnosed up to 30 days and from 30 days to 12 months after surgery, respectively. Delirium in non-surgical medical or intensive-care populations is referred to simply as delirium.

To make the strength of each statement explicit, we use three levels of support. A finding is described as *established* when it is supported by consistent randomized human evidence of at least moderate certainty; as *supported but unconfirmed* when it rests on human observational or physiological data, or on consistent *in vivo* animal data, without confirmation in randomized clinical studies; and as a *hypothesis* when it derives from a single model or laboratory, from cellular studies, or from mechanistic inference. The evidence underlying each principal claim is summarized in Table 1.

## 2 Molecular architecture of the circadian–melatonin axis

### 2.1 Core transcriptional–translational feedback loops

The mammalian clock comprises a primary negative-feedback loop and at least one interlocking stabilizing loop (Figure 1) (Takahashi, 2017). CLOCK and BMAL1 (ARNTL) heterodimerize and bind E-box and E′-box elements in the promoters of *Per1*, *Per2*, *Per3*, *Cry1*, and *Cry2*; accumulating PER and CRY proteins then multimerize, translocate to the nucleus, and repress CLOCK:BMAL1, closing the loop with a delay set by post-translational processing—principally casein kinase 1δ/ε-dependent phosphorylation, which governs PER stability and period length, with additional input from GSK-3β and from the AMPK–CRY and SIRT1–PER2 axes that couple the clock to cellular energy state (Takahashi, 2017; Hastings et al., 2018). In the stabilizing loop, CLOCK:BMAL1 drives *Nr1d1* (REV-ERBα) and *Rora*, whose products compete at RORE elements in the *Bmal1* promoter and impose antiphase *Bmal1* oscillation. CLOCK possesses intrinsic histone acetyltransferase activity, so clock function is inseparable from chromatin state—a point of direct relevance to anesthetic mechanism (Takahashi, 2017; Hastings et al., 2018; Doi et al., 2006; Akashi and Takumi, 2005)—while the NAD^+^-dependent deacetylase SIRT1 counter-regulates CLOCK-mediated acetylation, making NAD^+^ availability a metabolic input to clock amplitude (Nakahata et al., 2008; Nakahata et al., 2009).

**FIGURE 1:**
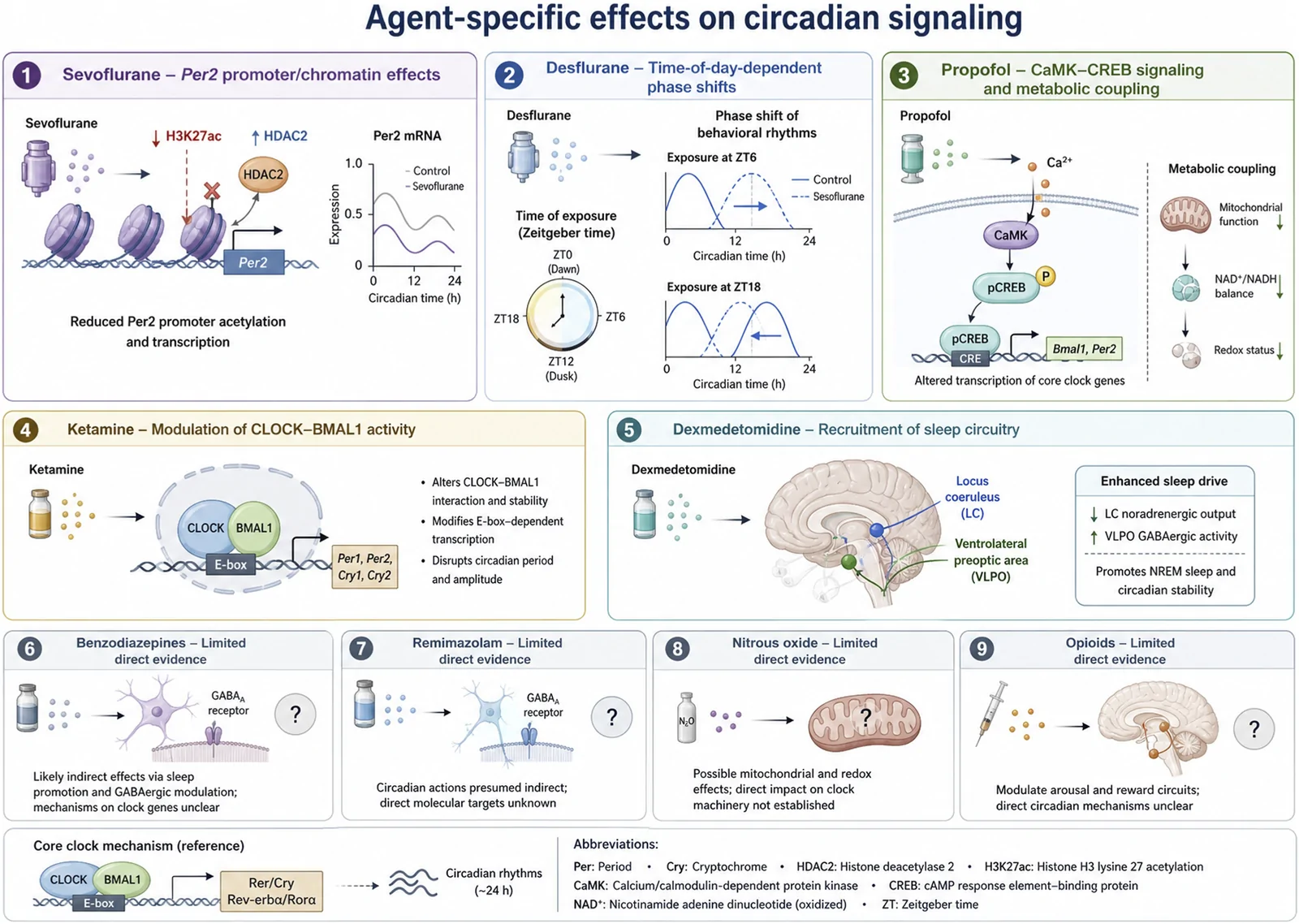
Molecular architecture of the circadian–melatonin axis. Integrated schematic showing retinal photic input to the SCN; CLOCK–BMAL1/PER–CRY and REV-ERB/ROR feedback loops; sympathetic control of pineal AANAT and ASMT; MT_1_/MT_2_ receptor signaling; and receptor-independent mitochondrial actions. Solid arrows denote established pathways; dashed arrows denote context-dependent or hypothesized perioperative interactions.

### 2.2 The suprachiasmatic nucleus and entrainment

The suprachiasmatic nucleus (SCN) contains approximately 20,000 coupled single-cell oscillators whose network synchrony—mediated largely by vasoactive intestinal peptide, arginine vasopressin, and GABAergic signaling—generates a pacemaker signal more precise and more robust than that of any constituent cell (Hastings et al., 2018). Photic entrainment proceeds through the retinohypothalamic tract: glutamate and PACAP release onto ventrolateral SCN neurons triggers NMDA receptor-dependent calcium influx, CaMKII and MAPK activation, CREB phosphorylation, and CRE-dependent induction of *Per1* and *Per2*, the molecular substrate of phase shifting (Hastings et al., 2018; Poulsen et al., 2018). Because this pathway is glutamatergic and because GABA is the dominant SCN transmitter, anesthetics acting as GABA_A_ positive allosteric modulators or NMDA receptor antagonists are pharmacologically positioned to interfere with entrainment itself rather than only with downstream rhythms (Poulsen et al., 2018; Ebling, 1996). Non-photic cues—arousal, locomotion, feeding, temperature, and melatonin—entrain principally through the intergeniculate leaflet and median raphe, with a phase–response relationship approximately antiphase to that of light (Poulsen et al., 2018; Slotten et al., 2005).

### 2.3 Melatonin biosynthesis and the efferent limb

The SCN drives pineal melatonin synthesis through a multisynaptic efferent pathway projecting sequentially to the paraventricular nucleus, the intermediolateral cell column, the superior cervical ganglion, and the pineal gland (Hastings et al., 2018; Brainard et al., 2001). Nocturnal noradrenaline release onto pinealocyte β_1_-and α_1_-adrenoceptors raises cAMP, activates PKA, and phosphorylates CREB, inducing and stabilizing arylalkylamine N-acetyltransferase (AANAT), the rate-limiting enzyme converting serotonin to N-acetylserotonin, which acetylserotonin O-methyltransferase then converts to melatonin (Brainard et al., 2001; Schomerus and Korf, 2005). Because AANAT is under acute adrenergic and photic control, with an action spectrum peaking near 460 nm, melatonin output is highly sensitive both to nocturnal light and to any perturbation of central sympathetic outflow (Brainard et al., 2001; Cajochen et al., 2005; Szewczuk et al., 2008). Anesthesia, sympatholysis, and operating-theatre or intensive-care illumination could therefore each suppress melatonin independently, which is one reason that perioperative melatonin suppression cannot safely be attributed to a single cause (Guo et al., 2023; Brainard et al., 2001).

### 2.4 Melatonin receptors and receptor-independent actions

MT_1_ (*MTNR1A*) and MT_2_ (*MTNR1B*) are class A G-protein-coupled receptors that couple preferentially to Gᵢ/o (Dubocovich et al., 2010; Jockers et al., 2016). Gαᵢ-mediated inhibition of adenylyl cyclase lowers cAMP, reduces PKA activity, and diminishes CREB phosphorylation, while liberated Gβγ activates G-protein-gated inwardly rectifying potassium channels and modulates calcium conductances; additional coupling to Gq/11–PLCβ, to MAPK/ERK, and, for MT_1_ in some cellular contexts, to Gs means that the direction of the cAMP response is context dependent rather than fixed. Both subtypes form homo- and heterodimers and heterodimerize with other receptors, including GPR50, with consequences for signaling efficiency (Zlotos et al., 2014), and structural studies have resolved their orthosteric pockets and a lipophilic ligand-access channel, providing a basis for subtype-selective drug design (Stauch et al., 2019; Johansson et al., 2019). Functionally the subtypes overlap but are not equivalent: in the SCN, MT_1_ contributes prominently to acute suppression of neuronal firing whereas MT_2_ has the larger role in phase shifting and chronobiotic signaling, so shifting circadian phase and reducing arousal are related but pharmacologically distinct uses of the melatonergic system (Dubocovich et al., 2010; Jockers et al., 2016).

Melatonin also has a putative binding site historically termed MT_3_, identified as quinone reductase 2, and has been proposed to interact with the nuclear receptor RORα, although the physiological importance of both remains contested (Jockers et al., 2016; Liu et al., 2019). Receptor-independent redox effects and modulation of antioxidant-enzyme systems have been described (Jockers et al., 2016; Reiter et al., 2016), and its amphipathic properties permit distribution into subcellular compartments including mitochondria, where experimental studies suggest local accumulation may occur (Reiter et al., 2016). This receptor-independent arm is considerably more exposure-dependent than high-affinity receptor signaling. Low-exposure chronobiotic treatment and high-exposure experimental cytoprotection therefore engage overlapping but non-equivalent mechanisms and should not be treated as interchangeable interventions.

Ramelteon, a selective MT_1_/MT_2_ agonist with higher receptor affinity than melatonin, low affinity for the MT_3_/quinone reductase 2 site, very low oral bioavailability, and an active metabolite (M-II) with a longer half-life (Kato et al., 2005; U.S. Food and Drug Administration, 2005), reproduces the receptor-mediated arm of melatonin pharmacology but has not been shown to share its receptor-independent redox or mitochondrial actions. In this review, “melatonin” and “ramelteon” therefore refer to the specific agents, and “melatonergic agents” is used only for pooled evidence or for statements that apply to both (Table 2).

### 2.5 Pharmacokinetics: formulation, dose, and exposure

Oral melatonin undergoes extensive first-pass hepatic metabolism, principally CYP1A2-mediated 6-hydroxylation followed by sulfate conjugation and urinary excretion (Lane and Moss, 1985). Mean absolute bioavailability of the immediate-release preparation was approximately 15% in a small pharmacokinetic study, with wide interindividual variability (DeMuro et al., 2000), and CYP1A2 activity is itself variable and modifiable: caffeine co-ingestion can substantially increase melatonin exposure, whereas smoking induces the enzyme and reduces it (Hartter et al., 2003). The elimination half-life of immediate-release melatonin is short, approximately 45 min, so a single preoperative dose is unlikely to maintain direct receptor occupancy across a delirium endpoint measured several days later (Lane and Moss, 1985; DeMuro et al., 2000); the same pharmacokinetic concern applies to analgesic trials that used a single dose 60–90 min before surgery (Oh et al., 2020).

Two implications follow. First, administered dose is an imprecise surrogate for systemic exposure, and perioperative inflammation, altered hepatic perfusion, and abrupt changes in smoking or caffeine intake may all modify CYP1A2 activity, yet these variables have rarely been measured in surgical trials (Lane and Moss, 1985; Hartter et al., 2003). Second, immediate-release, prolonged-release, sublingual, and intravenous formulations are not pharmacokinetically interchangeable: immediate-release preparations suit short-latency objectives such as preoperative anxiolysis or acute phase shifting, prolonged-release preparations are designed to sustain nocturnal exposure, and experimental cytoprotection generally requires substantially higher exposures (Lane and Moss, 1985; DeMuro et al., 2000).

## 3 Perioperative disruption of the circadian–melatonin axis

### 3.1 Amplitude suppression, phase delay, and rhythm loss

Human evidence is consistent in direction but heterogeneous in magnitude (Figure 2) (Gogenur et al., 2007a; Karkela et al., 2002). In 20 patients randomized to spinal or general anesthesia for minor orthopedic surgery, salivary melatonin secretion was significantly diminished on the first postoperative evening, nocturnal urinary 6-sulfatoxymelatonin excretion fell by approximately 26%, and the onset of nocturnal secretion was delayed (Karkela et al., 2002). Comparable disturbances of melatonin and core-temperature rhythms have been reported after both major and minimally invasive surgery, indicating that surgical magnitude alone does not determine circadian consequence (Gogenur et al., 2007a; Gogenur et al., 2007b), and the CLOCKS observational study has provided contemporary human data on sleep–wake timing after surgery and general anesthesia (van et al., 2022). Two limitations recur across this literature. Amplitude is measured more reliably than phase, because phase estimation demands frequent sampling under dim-light conditions that are rarely achievable in surgical patients. Anesthesia is also almost always confounded with surgical trauma, opioid exposure, fasting, environmental light, and psychological stress, so the human literature can establish that the axis is disrupted but seldom by what (Guo et al., 2023; Gogenur et al., 2007a; Karkela et al., 2002).

**FIGURE 2:**
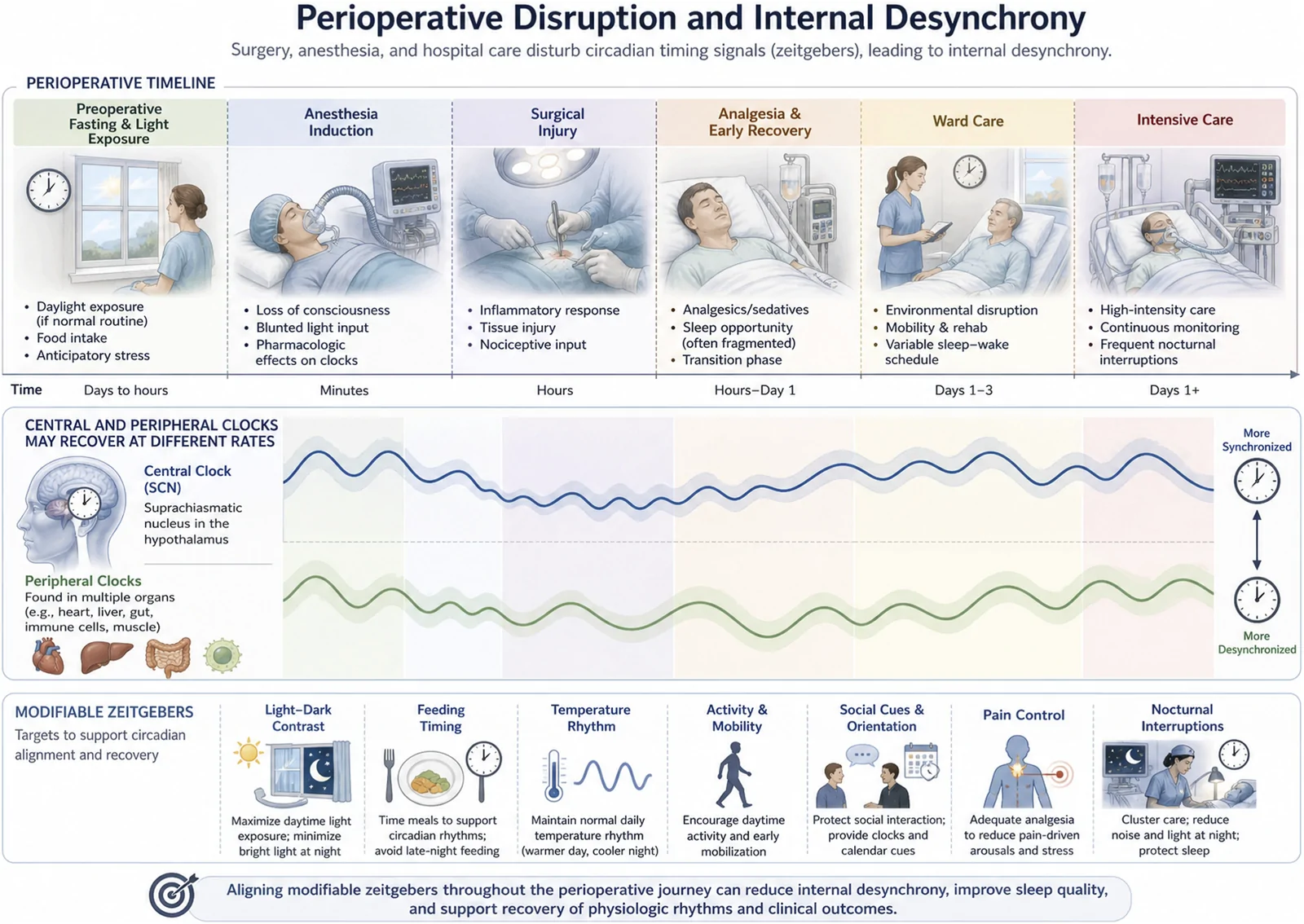
Perioperative disruption and internal desynchrony. Timeline from preoperative fasting and light exposure through anesthesia, surgical injury, analgesia, recovery, ward care, and intensive care. Central and peripheral clocks are shown as potentially recovering at different rates. Modifiable zeitgebers include light–dark contrast, feeding, temperature, activity, social cues, pain control, and nocturnal interruptions.

### 3.2 Anesthetic technique as a potential determinant

In a cohort of 138 older adults undergoing hip-fracture surgery in which plasma melatonin and cortisol were sampled every 6 h and delirium screened twice daily, general anesthesia was associated with a lower postoperative melatonin peak, mesor, and amplitude, more disturbed sleep, and a higher incidence of delirium; residual confounding by indication remains likely in this nonrandomized comparison (Song et al., 2021). By contrast, the randomized REGAIN trial found no reduction in delirium with spinal compared with general anesthesia (Neuman et al., 2021), arguing against a simple deterministic pathway running from anesthetic technique through melatonin suppression to delirium.

### 3.3 The intensive care environment

In sedated, mechanically ventilated patients the circadian melatonin rhythm is not merely blunted but can be abolished, and impaired rhythmicity has been documented in sedated critically ill patients with severe sepsis (Mekontso Dessap et al., 2025; Olofsson et al., 2004; Mundigler et al., 2002). The relevant exposure is not a discrete anesthetic event but a sustained composite of continuous sedation, absent light–dark contrast, round-the-clock nursing interventions, enteral feeding without circadian structure, systemic inflammation, and frequently catecholamine infusions that could plausibly interfere with adrenergic control of AANAT (Olofsson et al., 2004; Mundigler et al., 2002). The intensive care unit is thus simultaneously the setting in which circadian disruption is most extreme and the one in which mechanistic attribution is least tractable.

### 3.4 Melatonin as biomarker, mediator, or consequence

Preoperative cerebrospinal-fluid melatonin concentrations have been related to delirium in older patients with hip fracture, although that preliminary study was too small for firm inference (Scholtens et al., 2016). Three causal architectures remain compatible with the observed associations: melatonin suppression contributes causally to adverse outcomes; it marks an upstream driver such as inflammation, sympathetic activation, or surgical stress that independently causes the outcome; or suppression and outcome reinforce one another (Guo et al., 2023; Scholtens et al., 2016). Trials of exogenous melatonin cannot by themselves distinguish these possibilities, because a null result may reflect either non-causality or inadequate timing, formulation, or exposure (Lane and Moss, 1985; Scholtens et al., 2016). Causal inference would require triangulation across genetic-instrument approaches, mechanistic mediation studies, and trials that measure achieved plasma exposure and internal circadian phase rather than assuming them.

### 3.5 Environmental and behavioral desynchronizers

Perioperative care removes or mistimes several zeitgebers simultaneously. Patients may fast across the biological day, receive nutrition at night, remain immobile, and lose the normal day–night contrast in light, activity, social interaction, temperature, and feeding, while pain, nausea, catheters, drains, oxygen devices, and unfamiliar surroundings further fragment sleep (Olofsson et al., 2004; Mundigler et al., 2002). These exposures are modifiable, and they confound drug trials: a fixed 3 mg melatonin dose given in a brightly illuminated ward with frequent nocturnal interruptions tests a different biological intervention from the same dose embedded in a protected dark period, morning bright light, daytime mobilization, and appropriately timed feeding (Olofsson et al., 2004; Mundigler et al., 2002). The environmental schedule should therefore be treated as part of the intervention rather than as unmeasured background care.

## 4 Agent-specific effects on molecular clocks and melatonin

### 4.1 A framework of convergent mechanisms

Anesthetic effects on the clock can be organized around three non-mutually exclusive nodes whose relative importance varies across agents (Figure 3; Table 3) (Guo et al., 2023; Poulsen et al., 2018). The first is interference with entrainment: GABA_A_ positive allosteric modulation and NMDA receptor antagonism act on the two transmitter systems through which the SCN receives and integrates photic and non-photic time cues, so agents that potentiate GABAergic signaling in the SCN or attenuate glutamatergic retinohypothalamic transmission may disturb entrainment in addition to producing hypnosis (Poulsen et al., 2018). The second is transcriptional and epigenetic repression of core clock genes, principally direct suppression of *Per2* transcription in the SCN mediated by chromatin state and CLOCK–E′-box occupancy (Hastings et al., 2018; Poulsen et al., 2018). The third is metabolic coupling, through alterations in NAD^+^, SIRT1, AMPK, and GSK-3β activity that feed into post-translational control of PER and CRY stability (Takahashi, 2017; Hastings et al., 2018).

**FIGURE 3:**
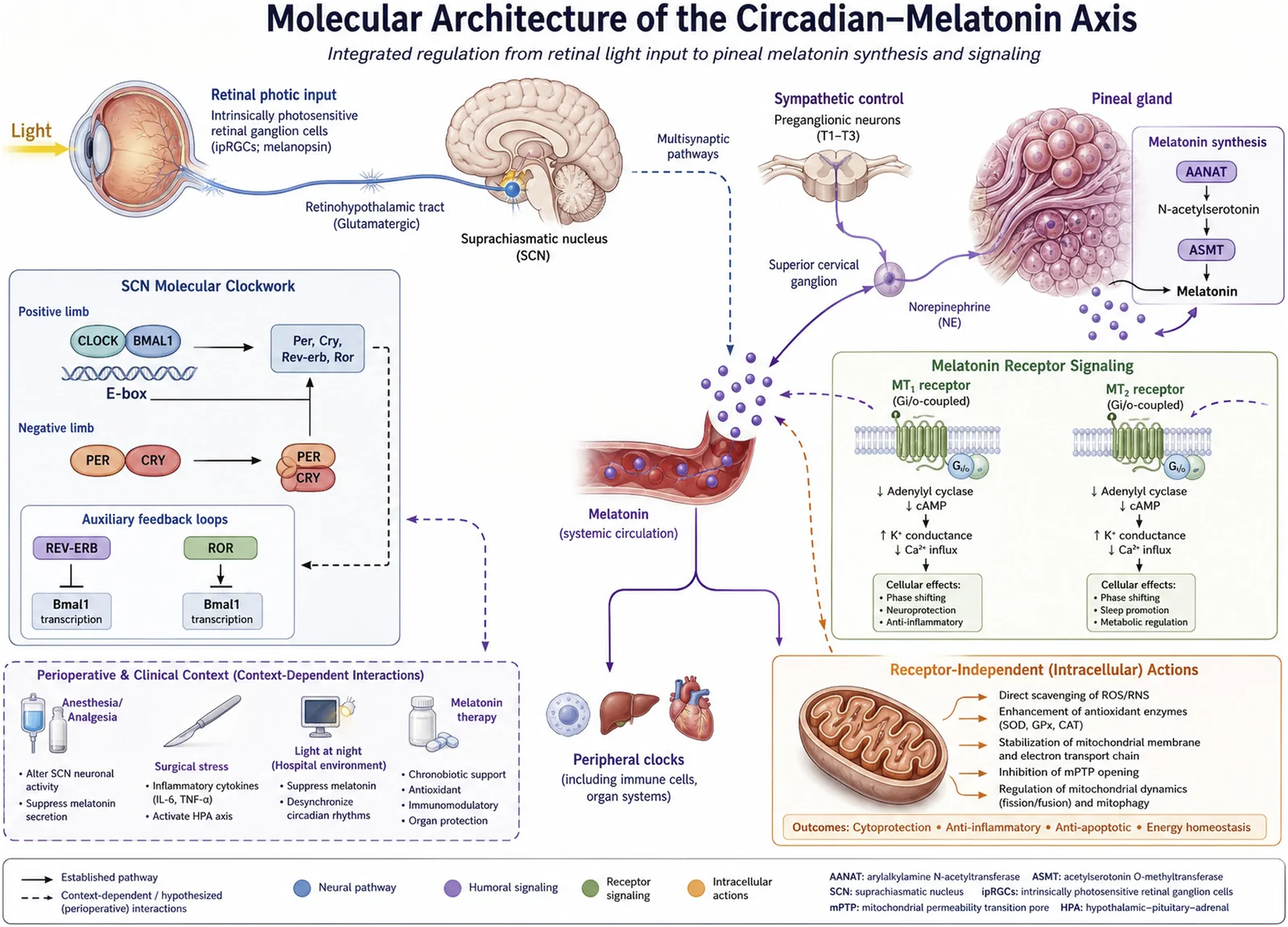
Agent-specific effects on circadian signaling. Comparative schematic of sevoflurane-associated reduction in CLOCK occupancy and histone H4 acetylation at the *Per2* promoter; desflurane time-of-day-dependent phase shifts; propofol CaMK–CREB and metabolic coupling; ketamine CLOCK–BMAL1 modulation; and dexmedetomidine recruitment of locus-coeruleus/ventrolateral-preoptic sleep circuitry. Direct circadian evidence for benzodiazepines, remimazolam, nitrous oxide, and opioids remains limited.

**TABLE 3:** Agent-specific interactions with the circadian–melatonin axis.

Agent	Principal targets/pathways	Clock-gene evidence	Phase or rhythm effect	Melatonin/sleep effect	Evidence level	References
Sevoflurane	GABA_A PAM; ↓CLOCK occupancy at *Per2* E′-box; ↓histone H4 acetylation at *Per2* promoter; NAD^+^ (contested)	Reversible repression of SCN *Per2* (∼64.5% of circadian expression); recovery after emergence; tissue-differential recovery kinetics	Phase delay of rest/activity rhythm; magnitude greatest with morning (rodent) exposure	No direct human rhythm evidence. In one rat day/night study, sevoflurane did not significantly change melatonin	*In vivo*, mouse and rat; consistent within one laboratory; no human data	*Per2* expression: (Ohe et al., 2011) Chromatin: (Mori et al., 2014) Phase: (Kadota et al., 2012) Melatonin: (Ocmen et al., 2016)
Isoflurane	GABA_A PAM	Suppression of clock genes in brain and liver (daytime exposure)	Time-dependent effects reported in rodents; clinical phase effect uncertain	No significant day/night melatonin change in one rat study; human melatonin-phase evidence remains unavailable	*In vivo*, mouse; no human data	Clock genes: (Gokmen et al., 2017) Comparative rhythm: (Sugimura et al., 2024) Melatonin: (Ocmen et al., 2016)
Desflurane	GABA_A PAM	Altered SCN *Per2*, *Bmal1* (*Arntl*), Clock, and *Cry1* mRNA	Phase shift depending on time of anesthetic exposure	Daytime melatonin increased in one rat study; human evidence is unavailable	*In vivo*, mouse; single study plus comparative study; no human data	Clock/phase: (Imai et al., 2020) Comparative rhythm: (Sugimura et al., 2024) Melatonin: (Ocmen et al., 2016)
Propofol	GABA_A PAM; CaMK–CREB signaling; GSK-3β/SIRT1/AMPK-linked metabolic regulation	↓hypothalamic Per, Cry	Human: desynchronization of rest–activity rhythm after light anesthesia for colonoscopy (low-trauma exposure)	↓oscillation amplitude of endogenous melatonin (rodent); melatonin pretreatment restores	Moderate (1 informative human study; consistent rodent)	Human rest–activity: (Dispe et al., 2009) Clock/melatonin rescue: (Yin et al., 2022)
Thiopental/benzodiazepines	GABA_A PAM	Not systematically characterized in SCN	Class-level nonphotic phase-shifting evidence is historical and preclinical; direct perioperative agent-specific evidence is sparse	Not established	Weak–moderate	Circadian class review: (Poulsen et al., 2018)
Remimazolam	GABA_A PAM (ultra-short-acting)	Insufficient direct evidence	Not established	Not established	Very limited	Current anesthesia–melatonin review: (Guo et al., 2023) No direct remimazolam phase study was identified
Ketamine	NMDA antagonist; BDNF–TrkB/mTOR	CLOCK–BMAL1 modulation shown in cellular models; perioperative relevance uncertain	Not established	Not established	Cellular models only; no human data	CLOCK–BMAL1 model: (Bellet et al., 2011)
Dexmedetomidine	α_2_-adrenoceptor agonist; locus coeruleus disfacilitation → VLPO disinhibition	Compared with propofol in rat brain; SCN effects incompletely defined	Not established in humans	Improves selected sleep outcomes; a human PK–PD study found no significant nocturnal melatonin change, and circadian resynchronization remains unproven	Moderate for sleep; insufficient for clock resynchronization	Brain clock comparison: (Yoshida et al., 2009) Sleep/PK–PD: (Akeju et al., 2018; Schnider et al., 2025)
Opioids	µ-opioid receptor	Not isolable in humans	Not established	Opioids can reduce slow-wave and REM sleep and contribute to sleep-disordered breathing; a direct melatonin-axis effect is not established	Weak (irreducible confounding)	Sleep architecture: (Wang and Teichtahl, 2007) Direct perioperative clock evidence remains insufficient

PAM, positive allosteric modulator; PK–PD, pharmacokinetic–pharmacodynamic; VLPO, ventrolateral preoptic nucleus. Evidence levels distinguish replicated preclinical findings from direct clinical phase evidence.

A fourth consideration cuts across all three nodes: phase dependence. The same agent, at the same dose and duration, may exert different clock effects according to circadian time (Guo et al., 2023; Poulsen et al., 2018). Mechanistic studies that omit zeitgeber time are correspondingly difficult to interpret, and clinical trials that do not record the time of exposure discard a variable that the underlying mechanism predicts to be important.

### 4.2 Sevoflurane

In rodents, sevoflurane has the most completely characterized molecular clock mechanism of any anesthetic, although the key studies all originate from one research group (Mori et al., 2014; Kadota et al., 2012; Ohe et al., 2011; Anzai et al., 2013; Matsuo et al., 2016). In mice, sevoflurane anesthesia suppressed circadian *mPer2* expression in the SCN by approximately 64.5%, with recovery after emergence (Ohe et al., 2011). No overt phase shift in *mPer2* expression was detected although the behavioral rhythm shifted after anesthesia, and a concurrent rise in suprachiasmatic NAD^+^ was proposed as the mediator of *mPer2* repression (Ohe et al., 2011). The effect is phase dependent: exposure to 2.5% sevoflurane in 40% oxygen for 4 h at different times of day produced the greatest repression of SCN *mPer2* after morning exposure (Kadota et al., 2012; Ohe et al., 2011), indicating that vulnerability to a standardized anesthetic exposure is not constant across the day (Kadota et al., 2012). A direct suprachiasmatic action is supported by studies combining quantitative *in situ* hybridization of rat *Per2 in vivo* with sevoflurane exposure of SCN explants from *Per2*-dLuc transgenic rats, in which explant bioluminescence was suppressed and phase delayed in the absence of systemic input (Anzai et al., 2013; Matsuo et al., 2016).

Chromatin immunoprecipitation of suprachiasmatic tissue from mice exposed to 2.5% sevoflurane in 40% oxygen for 1 or 4 h (Mori et al., 2014) showed that histone H4 acetylation at the proximal *Per2* promoter fell before *Per2* expression declined and that CLOCK binding to the *Per2* E′-box was concurrently reduced (Mori et al., 2014; Ohe et al., 2011). In that study suprachiasmatic NAD^+^ concentration was unchanged, and the authors concluded that reduced CLOCK occupancy and histone acetylation drove *Per2* repression independently of any detectable metabolic change (Mori et al., 2014; Ohe et al., 2011). The chromatin mechanism has therefore been observed consistently in studies from one laboratory, whereas the proposed NAD^+^ mediation was not reproduced; independent replication and human data are lacking, and translation to clinical anesthesia remains uncertain because the exposures used were species-, concentration-, and duration-specific (Mori et al., 2014; Ohe et al., 2011).

Circadian oscillators in different tissues recover at different rates after sevoflurane exposure, which suggests transient internal desynchrony rather than a uniform phase shift (Yamaguchi et al., 2022), and suppression of circadian gene expression has been reported to persist after awakening (Kobayashi et al., 2007). Central and peripheral clocks may therefore remain misaligned during early recovery (Yamaguchi et al., 2022; Kobayashi et al., 2007).

### 4.3 Isoflurane

Daytime isoflurane administration suppressed circadian gene expression in both brain and liver, indicating that volatile anesthetics engage central and peripheral oscillators (Gokmen et al., 2017), and a subsequent study compared sevoflurane, desflurane, and isoflurane directly in mice (Sugimura et al., 2024). Melatonin pretreatment attenuated isoflurane-associated cognitive dysfunction while modifying the sleep–wake rhythm in a murine model (Xia et al., 2016). This pharmacological rescue is consistent with, but does not establish, a causal role for circadian disruption; clock-gene manipulation or phase-specific human studies would be required to demonstrate that link.

### 4.4 Desflurane

In mice, desflurane produced a phase shift in running-wheel activity whose direction and magnitude depended on the time of administration (Sugimura et al., 2024; Imai et al., 2020) and altered SCN expression of *Per2*, *Bmal1* (*Arntl*), *Clock*, and *Cry1* (Imai et al., 2020). Its transcriptional effects thus extend beyond *Per2* to genes in both limbs of the clock while remaining phase dependent.

### 4.5 Propofol

Light propofol anesthesia for colonoscopy—an exposure without major surgical injury—was followed by desynchronization of the daily rest–activity rhythm over subsequent days, which reduces, although it does not eliminate, confounding by surgical trauma (Dispe et al., 2009). In rats, prolonged propofol anesthesia reduced the oscillation amplitude of sleep, body temperature, locomotor activity, and endogenous melatonin secretion over the following 24 h (Yin et al., 2022; Yoshida et al., 2009), inhibited hypothalamic *Per* and *Cry* expression, and implicated CaMK–CREB signaling together with the post-translational regulators GSK-3β, SIRT1, and AMPK (Yin et al., 2022; Yoshida et al., 2009). Melatonin pretreatment normalized the disrupted circadian phenotype, clock-factor expression, and the abnormal pineal melatonin response (Yin et al., 2022). Propofol and dexmedetomidine have also been compared directly for their effects on circadian gene expression in rat brain (Yoshida et al., 2009).

This pattern differs from the sevoflurane mechanism: whereas sevoflurane represses *Per2* through altered CLOCK occupancy and chromatin acetylation, propofol has been linked to CaMK–CREB and metabolic signaling (Yin et al., 2022). Because CaMK–CREB also participates in light-induced *Per1*/*Per2* expression, propofol could in principle act as a mistimed resetting stimulus rather than simply suppressing amplitude (Yin et al., 2022; Hastings et al., 2018; Dispe et al., 2009). This hypothesis is compatible with the observed rest–activity findings but remains unproven and requires human phase–response studies.

### 4.6 Thiopental, benzodiazepines, and remimazolam

Barbiturates and benzodiazepines are GABA_A_ positive allosteric modulators, and GABAergic signaling is central to suprachiasmatic network function (Poulsen et al., 2018); classical rodent work indicates that benzodiazepines can act as non-photic phase-shifting stimuli (Franks, 2008; Poulsen et al., 2018). Direct circadian evidence for remimazolam is at present insufficient. Its ultra-short pharmacokinetic profile makes it an informative comparator for longer-acting GABAergic agents, but available data support no claim that it either preserves or disrupts circadian function.

### 4.7 Ketamine and nitrous oxide

Ketamine is a non-competitive N-methyl-D-aspartate receptor antagonist with additional effects on opioid, monoaminergic, cholinergic, hyperpolarization-activated, BDNF–TrkB, and mTOR pathways (Hemmings et al., 2019; Bellet et al., 2011). In cellular models it altered CLOCK–BMAL1-dependent transcription and circadian gene expression through glycogen synthase kinase-3β-related signaling (Bellet et al., 2011). Its perioperative phase effects in humans remain uncharacterized, and antidepressant chronobiology cannot be assumed to apply at anesthetic doses. Nitrous oxide likewise inhibits N-methyl-D-aspartate signaling and may affect sleep indirectly through analgesia, opioid sparing, nausea, and arousal, but direct melatonin-rhythm evidence is lacking (Hemmings et al., 2019).

### 4.8 Dexmedetomidine

Dexmedetomidine is mechanistically distinctive because its sedative action recruits endogenous sleep-promoting circuitry: reduced locus coeruleus noradrenergic output disinhibits the ventrolateral preoptic nucleus (Akeju et al., 2018; Nelson et al., 2003), and in humans the drug can produce biomimetic non-rapid-eye-movement stage N3 sleep (Akeju et al., 2018). Low-dose nocturnal dexmedetomidine improved postoperative sleep quality in older intensive care patients (Nelson et al., 2003; Wu et al., 2016), and a randomized trial reported reduced delirium with nocturnal low-dose administration, whereas an intraoperative infusion strategy showed no benefit (Su et al., 2016; Deiner et al., 2017). Two caveats temper this interpretation. In a pharmacokinetic–pharmacodynamic study of subanesthetic oromucosal dexmedetomidine, nocturnal cortisol, melatonin, and heart rate did not change significantly despite measurable effects on sleep architecture (Schnider et al., 2025), supporting the view that sleep architecture and circadian phase are dissociable endpoints (Section 1.2). In addition, because α_2_-agonism reduces noradrenergic outflow whereas pineal AANAT is under adrenergic control, an effect on melatonin synthesis is plausible but has not been adequately tested during the sustained infusions used perioperatively (Schnider et al., 2025; Nelson et al., 2003).

### 4.9 Opioids and multimodal adjuncts

Opioids commonly fragment sleep architecture and suppress slow-wave and rapid-eye-movement sleep during the perioperative period (Andersen et al., 2014; Wang and Teichtahl, 2007). Because opioid exposure covaries with pain, surgical magnitude, and anesthetic technique, its independent circadian contribution is difficult to isolate, and opioid dose and timing should be treated as important potential confounders in perioperative circadian studies (Olofsson et al., 2004; Mundigler et al., 2002).

### 4.10 Surgical trauma, cytokines, and the HPA axis

Anesthesia is only one perioperative input to the clock. Surgical trauma triggers an acute-phase response with increases in IL-6, IL-1β, and TNF-α; inflammatory signaling can alter clock-gene expression in peripheral tissues and brain; cortisol, an important peripheral zeitgeber, loses its normal rhythmicity after major surgery; and pain, fasting, immobilization, and psychological stress supply further non-photic input (Gogenur et al., 2007a; Hastings et al., 2018). The anesthetic contribution to human chronodisruption is therefore inferred largely from animal studies performed without surgery (Mori et al., 2014; Ohe et al., 2011) and from low-trauma human procedures such as propofol sedation for colonoscopy (Dispe et al., 2009).

### 4.11 Circadian modulation of anesthetic pharmacodynamics

The relationship is bidirectional. In mice, hypnotic effects on recovery from isoflurane anesthesia varied with the light–dark cycle, and the chronopharmacological effect of suvorexant was lost in blind animals, suggesting that intact daily light–dark cues contribute to the time-dependent response (Sugano et al., 2021). In humans, circadian variation in the Narcotrend index and in propofol target-controlled infusion concentrations has been reported, and time-of-day effects on elective-surgery outcomes have been systematically reviewed (Meewisse et al., 2024; Shen et al., 2021). Whether depth-of-anesthesia targets and infusion regimens used at 08:00 are pharmacodynamically equivalent to those used at 16:00 remains clinically relevant but unresolved.

## 5 Melatonergic signaling and perioperative organ protection: predominantly preclinical evidence

Unless stated otherwise, the evidence in this section comes from animal and cellular models; no adequately powered human trial has shown that melatonin protects any organ during surgery.

### 5.1 Neuroinflammation, NLRP3, and synaptic vulnerability

Rodent models of anesthesia- and surgery-associated cognitive impairment implicate hippocampal microglial NLRP3 inflammasome activation and IL-1β release (Figure 4) (Yang et al., 2024; Zhu et al., 2023). In mice, sevoflurane-associated cognitive dysfunction has been linked to mitochondrial DNA release and cGAS–STING-dependent NLRP3 activation in microglia, while surgical trauma induces age-dependent ASC-speck formation in CA1 microglia (Yang et al., 2024; Zhang et al., 2022). In rodent models, melatonin reduced NLRP3–caspase-1–IL-1β signaling and NF-κB phosphorylation and nuclear translocation in models of postoperative cognitive dysfunction (Zhu et al., 2023), and in aged mice undergoing laparotomy with sevoflurane it improved postoperative sleep and neurobehavioral outcomes while modifying SCN clock-gene rhythmicity (Jia et al., 2024). Whether either mechanism operates at the plasma concentrations achieved with 3–5 mg oral melatonin in older surgical patients is uncertain, because preclinical studies generally use much higher weight-adjusted exposures (Yang et al., 2024; Zhu et al., 2023).

**FIGURE 4:**
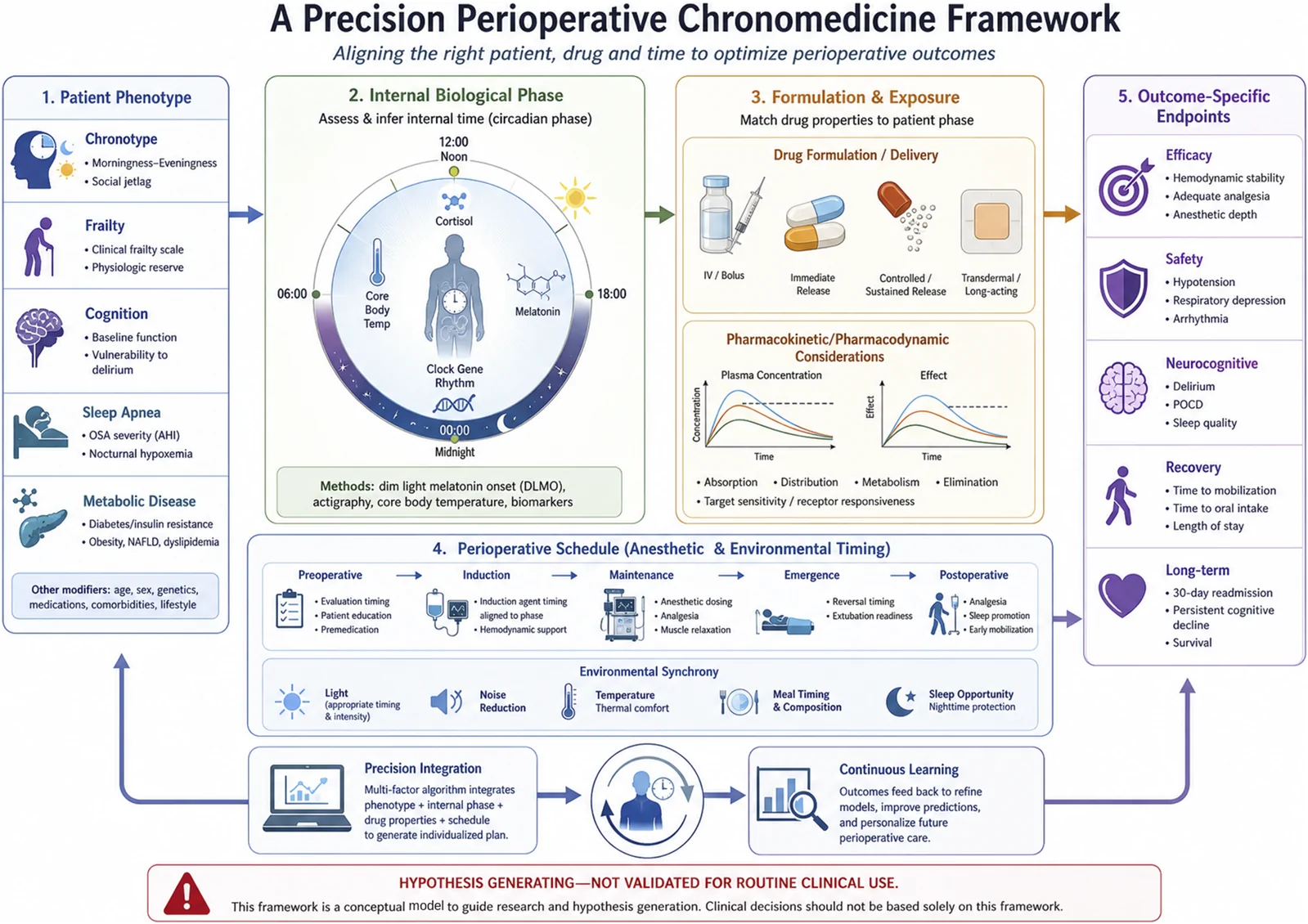
Melatonin-linked pathways relevant to perioperative organ injury. MT1/MT2 receptor pathways and receptor-independent actions converge on mitochondrial permeability transition, fission–mitophagy balance, NF-κB/NLRP3 neuroinflammation, endothelial signaling, and ischemia–reperfusion injury. Most organ-protection mechanisms shown are derived from animal and cellular studies and have not been confirmed in surgical patients (Table 1). A separate REV-ERB branch emphasizes that circadian cardioprotection is not necessarily melatonin mediated.

### 5.2 Mitochondrial cardioprotection and the REV-ERB paradigm

On-pump cardiac surgery offers a controlled human model of ischemia–reperfusion. In isolated perfused rat hearts, melatonin attenuated ischemia–reperfusion injury, an effect attributed at least partly to inhibition of mitochondrial permeability transition pore opening: after global ischemia–reperfusion it improved functional recovery, reduced infarct size and lactate dehydrogenase release, and rendered isolated mitochondria more resistant to calcium-induced pore opening (Petrosillo et al., 2009). Whether this occurs in patients undergoing cardiac surgery has not been tested in adequately powered trials. More granular mechanisms have since been described, including AMPKα-dependent suppression of Drp1-mediated mitochondrial fission, preservation of the VDAC1–hexokinase 2 interaction, and limitation of excessive mitophagy in cardiac microvascular endothelial cells (Zhou et al., 2017; Wang et al., 2020).

In a study of 596 propensity-matched patients undergoing aortic valve replacement, the incidence of major adverse cardiac events over 500 days was lower after afternoon than after morning surgery (hazard ratio 0.50, 95% CI 0.32–0.77; p = 0.0021), and in a randomized substudy of 88 patients perioperative myocardial injury measured by cardiac troponin T release was lower in the afternoon group (ratio of geometric means 0.79, 95% CI 0.68–0.93; p = 0.0045) (Montaigne et al., 2018). Transcriptomic analysis of myocardial samples implicated the circadian nuclear receptor REV-ERBα, and in a mouse Langendorff hypoxia–reoxygenation model *Rev-Erbα* deletion or antagonist treatment reduced injury at the sleep-to-wake transition through increased expression of CDKN1a/p21 (Montaigne et al., 2018). These findings have not been consistently replicated for hard clinical outcomes: neither a diverse multi-institutional cardiac-surgery network nor a propensity-matched analysis of afternoon aortic-valve surgery detected an outcome effect of time of day (Nemeth et al., 2021; Gotte et al., 2020); an updated Cochrane review likewise does not support routine chronoscheduling of cardiac surgery (Liu et al., 2026). Experimental evidence therefore supports circadian regulation of myocardial ischemic tolerance, but the clinical proposition that rescheduling cardiac surgery improves outcomes is not established (Meewisse et al., 2024; Montaigne et al., 2018), and the pathway identified is REV-ERBα-dependent rather than melatonin-dependent.

### 5.3 Hepatic and other-organ ischemia–reperfusion

Melatonin attenuated hepatic ischemia–reperfusion injury through the PGAM5–mPTP pathway in a recent preclinical model, and related mitochondrial mechanisms have been described in other organs (Shi et al., 2024; Tan et al., 2016). Potential clinical settings include liver resection with vascular occlusion and transplantation, but efficacy in human hepatectomy, renal injury, or pulmonary ischemia–reperfusion has not been established in adequately powered trials.

### 5.4 Anesthetic-sparing and antinociceptive actions

In a prospective randomized double-blind dose–response study of 200 ASA I adults, oral melatonin 0.2 mg/kg given approximately 50 min before induction reduced the propofol ED_50_ for loss of response to verbal command from 1.5 mg/kg (95% CI 1.4–1.6) to 0.9 mg/kg (0.8–0.96) and the thiopental ED_50_ from 3.4 mg/kg (3.2–3.5) to 2.7 mg/kg (2.6–2.9) (Naguib et al., 2006). Oral melatonin 3 or 5 mg also reduced the propofol dose required to reach a bispectral index of 45 without prolonging recovery-room stay (Turkistani et al., 2007). Effects of this magnitude warrant independent replication with contemporary monitoring and measured melatonin concentrations (Naguib et al., 2006; Turkistani et al., 2007). MT_1_-mediated reduction of neuronal excitability offers a plausible mechanism, but the clinical interaction has not been linked directly to a specific receptor pathway.

### 5.5 Redox balance and dose–mechanism separation

Melatonin is frequently described as an antioxidant, but perioperative redox biology is compartmentalized and time-dependent, and reactive oxygen species also participate in host defense, vascular signaling, mitochondrial adaptation, and wound repair, so indiscriminate suppression is not necessarily beneficial. As noted in Section 2.4, low-dose receptor-mediated treatment cannot be assumed to reproduce the intracellular concentrations used for high-dose cytoprotection (Reiter et al., 2016; Tan et al., 2016); dose and formulation should be matched to the intended mechanism and paired with compartment-relevant biomarkers such as F2-isoprostanes, glutathione redox state, mitochondrial respiration, or validated transcriptional signatures.

## 6 Comorbidity and host factors as effect modifiers

### 6.1 Aging, frailty, and neurodegenerative vulnerability

Endogenous melatonin amplitude often declines with age, although interindividual variability is substantial (Table 4) (Verma et al., 2023; Zitting and Yuan, 2025). Older adults may therefore enter surgery with lower circadian amplitude and reduced physiological reserve, but lower amplitude does not imply greater treatment responsiveness. No perioperative trial has stratified melatonin effects by baseline circadian amplitude, and the possibility that impaired SCN or downstream circuitry reduces responsiveness remains untested.

**TABLE 4:** Comorbidities and host factors that may modify the axis.

Factor	Effect on axis	Mechanistic basis	Potential implication	Evidence status	References
Aging	The amplitude of nocturnal melatonin secretion generally decreases with age, although interindividual variability and preserved rhythms in some older adults are substantial	Age-related alterations in SCN output, retinal light input, pineal responsiveness, sleep timing, and entrainment	Baseline circadian phase and melatonin amplitude should be measured because treatment effects may differ between individuals	Human physiological association; perioperative treatment interaction untested	Updated human circadian-aging review (2025): (Zitting and Yuan, 2025)
Dementia/MCI	Circadian disturbance and sundowning are common in dementia; pre-existing cognitive impairment is an established POD risk factor	Neurodegeneration may impair the SCN and downstream circadian circuitry, but the pathway to POD is multifactorial	Direction of treatment-effect modification remains unknown; existing trials are underpowered for interaction testing	Associations established; melatonin-treatment interaction untested	Updated sundowning review (2025): (Reimus and Sieminski, 2025) Recent POD risk synthesis/model (2025): (Wan et al., 2025)
*MTNR1B* rs10830963 G risk allele	Enhanced melatonin-receptor signaling is associated with impaired early insulin secretion and increased type 2 diabetes risk	Higher *MTNR1B* expression in human islets and MT2-mediated inhibition of glucose-stimulated insulin secretion	Possible genotype-dependent glycemic response; perioperative clinical relevance remains untested	Strong human genetic and mechanistic evidence; perioperative interaction untested	Foundational genetic association: (Lyssenko et al., 2009) Human islet and signaling validation: (Tuomi et al., 2016)
Type 2 diabetes/obesity/insulin resistance	Melatonin timing and *MTNR1B* genotype may worsen glucose tolerance; surgical stress independently increases hyperglycemia risk	MT2-mediated inhibition of beta-cell insulin secretion plus stress-hormone-driven insulin resistance	Prespecified perioperative glucose monitoring is appropriate in melatonin trials, particularly in patients with diabetes or obesity	Human non-surgical interaction evidence; surgical safety interaction untested	Human genotype-melatonin glucose interaction: (Garaulet et al., 2015) Human islet and signaling study: (Tuomi et al., 2016)
Cardiovascular disease/beta1-blocker use	Beta1-adrenergic blockade can suppress nocturnal melatonin secretion in humans	Sympathetic beta1-adrenergic input contributes to pineal melatonin synthesis; blockade can reduce this signal	Baseline melatonin rhythm and replacement response may differ; clinical relevance remains untested	Direct but small human pharmacology evidence; perioperative interaction untested	Direct atenolol dose-response study: (Nathan et al., 1997)
Obstructive sleep apnea	OSA is associated with sleep fragmentation, intermittent hypoxia, sympathetic activation, and altered melatonin secretion profiles	Intermittent hypoxia, sleep fragmentation, and circadian-autonomic dysregulation	OSA may modify sleep, respiratory, and neurocognitive outcomes; the net perioperative melatonin effect is uncertain	Association supported; perioperative efficacy and respiratory-safety interactions remain insufficiently tested	24-h melatonin-profile study (2024): (Karel et al., 2024) Updated OSA sleep-wake review (2025): (Fernandes and Liguori, 2025)
Hepatic impairment	Liver disease may alter endogenous melatonin rhythms and oral exposure, but direct melatonin pharmacokinetic data in hepatic impairment are limited	Extensive first-pass hepatic extraction; metabolism occurs principally through CYP1A2	Fixed oral dosing may yield variable or increased exposure; no validated hepatic dose-adjustment rule is available	Indirect pharmacokinetic rationale; hepatic-impairment dosing evidence is insufficient	First-pass hepatic metabolism study: (Lane and Moss, 1985) Systematic clinical pharmacokinetic review: (Harpsoe et al., 2015)
CYP1A2 variability/phenoconversion	CYP1A2 induction or inhibition can decrease or increase melatonin exposure; direct interactions are best established for caffeine and fluvoxamine	CYP1A2-mediated 6-hydroxylation is a principal clearance pathway; inflammation and smoking changes may alter enzyme activity	Medication, smoking, caffeine, and inflammatory status should be recorded because a fixed oral dose does not ensure fixed exposure	Established human pharmacokinetic interactions; rarely measured perioperatively	Direct pharmacokinetic interactions: caffeine (Hartter et al., 2003); fluvoxamine (Hartter et al., 2000)
Chronotype/shift work/induction time	These factors determine the internal circadian phase at anesthesia induction	The effect of volatile anesthetics on circadian timing depends on anesthetic type, clock time, and phase relationship	Chronotype and clock time may modify circadian disruption and should be prespecified in perioperative trials	Time-of-day dependence demonstrated in rodents; limited human perioperative rhythm data; clinical interaction untested	Desflurane phase dependence in mice (Imai et al., 2020); comparative volatile-anesthetic rhythm study (Sugimura et al., 2024)
Habitual melatonin/ramelteon use; blindness	Prior melatonergic exposure may alter baseline response; total blindness can produce a free-running circadian phase	Prior receptor-agonist exposure and absent or reduced photic entrainment	These factors may alter baseline phase, exposure, and treatment interpretation	Non-24-h disorder is established in blindness; the perioperative treatment interaction remains untested	Updated Non-24-h sleep-wake disorder review: (Abbott, 2019) No direct perioperative interaction study identified

AUC, area under the curve; MCI, mild cognitive impairment; OSA, obstructive sleep apnea; PK, pharmacokinetics; POD, postoperative delirium; SCN, suprachiasmatic nucleus. Predicted treatment implications are not validated clinical rules.

### 6.2 Pre-existing neurocognitive disorder

Dementia and mild cognitive impairment are among the strongest risk factors for postoperative delirium and are frequently accompanied by circadian disturbance, including sundowning (Evered et al., 2018; de Jonghe et al., 2014; Reimus and Sieminski, 2025; Wan et al., 2025); more than half of the participants in the largest hip-fracture melatonin trial had cognitive impairment (de Jonghe et al., 2014). Whether such patients are more responsive because circadian pathology is greater, or less responsive because central clock circuitry is impaired, remains unresolved, and available trials are not powered to test this interaction.

### 6.3 Diabetes, obesity, and *MTNR1B*

The common intronic variant rs10830963 in *MTNR1B* is associated with fasting glucose and type 2 diabetes risk (Lyssenko et al., 2009). The G risk allele increases *MTNR1B* expression in human islets and can amplify melatonin-mediated inhibition of insulin secretion through Gi-coupled MT_2_ receptors (Lyssenko et al., 2009; Tuomi et al., 2016). In a small experimental study of 17 normoglycemic women, the glycemic response to 5 mg melatonin differed by genotype, and the variant explained a substantial proportion of interindividual variation in the glucose area-under-the-curve response (Garaulet et al., 2015). These non-surgical data support a pharmacogenomic safety hypothesis but do not establish a need for routine perioperative genotyping. Because diabetes, stress hyperglycemia, meal timing, fasting, insulin therapy, and corticosteroids may all modify any glycemic effect of exogenous melatonin, trials in metabolically vulnerable patients should prespecify glucose monitoring and genotype-stratified exploratory analyses rather than assume either benefit or harm (Lyssenko et al., 2009; Tuomi et al., 2016).

### 6.4 Cardiovascular disease, heart failure, and obstructive sleep apnea

β_1_-adrenergic blockers can suppress nocturnal melatonin secretion by attenuating adrenergic induction of AANAT (Nathan et al., 1997), and obstructive sleep apnea adds sleep fragmentation, intermittent hypoxia, and sympathetic activation and has been associated with altered 24-h melatonin profiles (Karel et al., 2024). Whether exogenous melatonin improves or worsens perioperative respiratory and neurocognitive outcomes in untreated severe obstructive sleep apnea remains insufficiently studied.

### 6.5 Hepatic dysfunction and CYP1A2 variability

Because melatonin undergoes substantial hepatic first-pass clearance, cirrhosis can alter both endogenous rhythms and the disposition of exogenous drug (Lane and Moss, 1985). Inflammation may downregulate CYP1A2, perioperative smoking cessation removes an inducer, and caffeine withdrawal removes an inhibitor; the combined direction and magnitude of these changes in an individual patient are not reliably predictable, so a fixed 3 mg oral dose represents a fixed administered dose rather than a fixed biological exposure (Lane and Moss, 1985; Hartter et al., 2003).

### 6.6 Chronotype, shift work, blindness, and habitual melatonin use

Chronotype determines the phase relationship between a patient’s internal clock and the operating schedule: an extreme evening type undergoing early-morning surgery is exposed at a different internal circadian time from an extreme morning type, and rodent data indicate that internal time can modify anesthetic clock effects (Kadota et al., 2012). Free-running blind patients, shift workers, and patients already taking melatonin or ramelteon are commonly excluded, unrecorded, or too few for analysis, and although chronotype questionnaires such as the MEQ or MCTQ are inexpensive, chronotype has rarely been incorporated systematically into perioperative melatonin trials (Kadota et al., 2012; Sugano et al., 2021; Abbott, 2019).

### 6.7 Cancer, immune context, and oncologic surgery

Cancer and anticancer therapy can disrupt circadian organization through cachexia, corticosteroids, chemotherapy, hospitalization, nocturnal symptoms, and altered activity or feeding schedules (Guo et al., 2023; Reiter et al., 2016; Counts et al., 2020; Ma et al., 2021). Melatonin has been investigated in oncology, but current perioperative evidence does not establish anticancer efficacy. Trials in oncologic surgery should separate short-term sleep or delirium outcomes from recurrence, immune competence, treatment interactions, and patient-reported recovery, which require larger cohorts and longer follow-up.

## 7 Clinical outcomes: a critical appraisal

### 7.1 Preoperative anxiety and sedation

A Cochrane review of 27 randomized trials and 2,319 participants found that, compared with placebo, melatonin probably reduced preoperative anxiety (mean difference −11.69 mm on a 0–100 mm visual analogue scale; 95% CI −13.80 to −9.59; 18 studies, 1,264 participants; moderate-certainty evidence), and that compared with benzodiazepines it probably produced little or no difference (mean difference 0.78 mm; 95% CI −2.02 to 3.58; 7 studies, 409 participants) (Table 5) (Madsen et al., 2020). Reported adverse effects were generally mild and no serious treatment-related adverse event was identified.

**TABLE 5:** Representative randomized and pooled clinical evidence.

Study/design	Population	Intervention	Primary outcome	Main finding	Interpretation
Madsen et al., 2020 Cochrane SR/MA (Madsen et al., 2020)	27 RCTs; n = 2,319 adult surgical patients	Melatonin 3–10 mg or 0.05–0.4 mg/kg vs. placebo or benzodiazepine	Preoperative and immediate postoperative anxiety (VAS 0–100 mm)	Vs. placebo: MD −11.69 mm (95% CI −13.80 to −9.59; 18 studies, n = 1,264; moderate certainty). Vs. benzodiazepines: MD 0.78 mm (−2.02 to 3.58; 7 studies, n = 409; moderate certainty)	Melatonin modestly reduces preoperative anxiety vs. placebo and may be comparable with benzodiazepines; the immediate postoperative effect was smaller and of low certainty
Bolt et al., 2024 MAGIC RCT (Bolt et al., 2024)	110 anxious children across 20 UK NHS trusts; trial stopped early	Melatonin 0.5 mg/kg (max 20 mg) vs. midazolam 0.5 mg/kg (max 20 mg)	Modified Yale Preoperative Anxiety Scale–Short Form (mYPAS-SF)	Adjusted MD 13.1 points (95% CI 3.7–22.4), favoring midazolam; the noninferiority criterion was not met.	Melatonin was less effective than midazolam. Early termination reduced precision but did not support noninferiority
Hatta et al., 2014 RCT (DELIRIA-J) (Hatta et al., 2014)	67 adults aged 65–89 years admitted for acute care (24 ICU; 43 wards)	Ramelteon 8 mg nightly for up to 7 days vs. placebo	Delirium diagnosed using DSM-IV criteria	Delirium: 3% vs. 32% (p = 0.003); adjusted OR 0.07 (95% CI 0.008–0.54). ARR 29%; derived NNT approximately 4	Large apparent effect, but the sample was small and the confidence interval wide; confirmation in larger medical cohorts was required
de Jonghe et al., 2014 MAPLE RCT (de Jonghe et al., 2014)	452 randomized; 378 analyzed; patients ≥65 years with acute hip fracture (mean age 84 years)	Melatonin 3 mg nightly for 5 days vs. placebo	Incident delirium within 8 days	Melatonin did not reduce delirium incidence	A large, rigorous negative trial in an elderly hip-fracture population; post-randomization exclusions should be considered when interpreting generalizability
Kinouchi et al., 2023 RCT (Kinouchi et al., 2023)	Older patients undergoing surgery under general anesthesia	Ramelteon vs. placebo	Postoperative delirium (POD)	No significant difference in POD incidence between groups	Does not support routine ramelteon prophylaxis after general anesthesia
Oh et al., 2021 RECOVER RCT (Oh et al., 2021)	Older adults undergoing elective primary or revision hip or knee replacement	Ramelteon 8 mg nightly vs. placebo	Postoperative delirium	Ramelteon was not more effective than placebo for preventing POD.	A negative orthopedic-surgery trial, consistent with other perioperative ramelteon studies
Ford et al., 2020 Healthy Heart–Mind RCT (Ford et al., 2020)	Adults undergoing major cardiac surgery	Melatonin vs. placebo	Postoperative delirium	Melatonin did not prevent delirium after major cardiac surgery	Does not support prophylactic melatonin in this cardiac-surgery setting
Lange et al., 2024 RCT (Lange et al., 2024)	120 medical inpatients ≥65 years with established hyperactive or mixed delirium	Immediate-release melatonin 5 mg nightly for 5 nights vs. placebo	Change in Memorial Delirium Assessment Scale (MDAS)	Mean MDAS improvement: 4.9 vs. 5.4 points (p = 0.42); no significant reduction in delirium severity	This was a treatment—not prevention—trial and did not support melatonin for reducing established delirium severity
Han et al., 2020 SR/MA (Han et al., 2020)	6 RCTs plus 3 observational studies	Melatonin or ramelteon	Postoperative delirium	Overall OR 0.45 (95% CI 0.24–0.84; p = 0.01). Timing subgroup (<5 elimination half-lives before surgery): OR 0.31 (0.19–0.49)	The pooled signal was favorable, but inclusion of observational studies and subgroup analyses limits causal certainty
Shin et al., 2024 SR/MA (Shin et al., 2024)	16 RCTs of surgical patients	Perioperative melatonin or melatonin-receptor agonists	POD and adverse events	Pooled analysis suggested lower POD incidence, with stronger subgroup signals for higher-dose melatonin and cardiopulmonary surgery	Clinical, formulation, timing, and methodological heterogeneity make subgroup findings hypothesis-generating
Borges et al., 2026 SR/MA (Borges et al., 2026)	16 RCTs in surgical patients >60 years (12 melatonin; 4 ramelteon)	Perioperative melatonin or ramelteon vs. placebo/nonpharmacological control	Postoperative delirium	Overall RR 0.72 (95% CI 0.56–0.92; p = 0.009); melatonin RR 0.67 (0.52–0.88); ramelteon RR 0.98 (0.47–2.01); ≥5 mg subgroup RR 0.52 (0.40–0.68)	The most recent perioperative pairwise meta-analysis supports a possible melatonin-specific and dose-related effect, but certainty remains limited by heterogeneity
Queiroz et al., 2026 Bayesian NMA (Queiroz et al., 2026)	79 RCTs; 24,827 patients undergoing cardiac surgery	29 perioperative pharmacological strategies	Postoperative delirium	Dexmedetomidine plus melatonin ranked highly vs. placebo (RR 0.31; 95% credible interval 0.13–0.69)	Low-certainty network estimate based substantially on indirect comparisons; direct head-to-head replication is needed
Chaves-Filho et al., 2026 updated SR/MA (Chaves-Filho et al., 2026)	24 ICU RCTs; n = 3,680 (primary placebo-controlled analysis: 18 studies)	Melatoninergic agonists (melatonin or ramelteon) vs. placebo	ICU delirium; mortality; length of stay; mechanical ventilation; safety	Delirium RR 0.77 (95% CI 0.62–0.94). Melatonin subgroup RR 0.77 (0.62–0.97); surgical ICU RR 0.64 (0.48–0.87). No mortality benefit; other secondary outcomes were inconclusive	Most recent ICU synthesis; modest effect based on low-certainty evidence with moderate heterogeneity

Abbreviations: ARR, absolute risk reduction; BZD, benzodiazepine; CI, confidence interval; CrI, credible interval; MD, mean difference; MV, mechanical ventilation; NMA, network meta-analysis; NNT, number needed to treat; POD, postoperative delirium; RCT, randomized controlled trial; RR, risk ratio; SR/MA, systematic review/meta-analysis; VAS, visual analogue scale.

The pediatric evidence requires separate interpretation. In the multicenter double-blind MAGIC noninferiority trial conducted across 20 UK NHS trusts, anxious children received melatonin or midazolam (both 0.5 mg/kg, maximum 20 mg); the adjusted mean difference on the modified Yale Preoperative Anxiety Scale–Short Form favored midazolam by 13.1 points (95% CI 3.7–22.4), and noninferiority of melatonin was not demonstrated (Bolt et al., 2024). Recruitment stopped early for futility at 110 of a planned 624 participants, which reduces precision, but the available evidence does not support extrapolating adult equivalence to children. Preoperative anxiolysis nonetheless remains the best-supported perioperative use of melatonin in adults, with moderate-certainty evidence and generally mild short-term adverse effects (Madsen et al., 2020; Bolt et al., 2024).

### 7.2 Postoperative delirium

The delirium literature is inconsistent, and its heterogeneity is mechanistically informative. Because melatonin and ramelteon differ in pharmacology and evidence base (Table 2), trials of each agent are considered separately, and surgical trials are distinguished from those in medical or intensive-care populations (Section 1.2).

#### 7.2.1 Melatonin in surgical patients

In the largest trial, 452 patients aged 65 years or older undergoing acute hip surgery were randomized to melatonin 3 mg or placebo for 5 consecutive days; 378 were analyzed after postrandomization exclusion (mean age 84 years; 55.6% with cognitive impairment), and postoperative delirium was not reduced (de Jonghe et al., 2014). Melatonin also showed no benefit after major cardiac surgery in the Healthy Heart–Mind trial (Ford et al., 2020), whereas a smaller trial in hip-arthroplasty patients reported benefit (Sultan, 2010). In pooled analyses restricted to melatonin, the most recent meta-analysis reported a risk ratio of 0.67 (95% CI 0.52–0.88), with a stronger signal at doses of 5 mg or more (Borges et al., 2026).

#### 7.2.2 Ramelteon in surgical patients

Ramelteon was not effective in preventing postoperative delirium after general anesthesia in older patients (Kinouchi et al., 2023) or after orthopedic surgery in the RECOVER trial (Oh et al., 2021), and the pooled ramelteon estimate in surgical trials was null (risk ratio 0.98, 95% CI 0.47–2.01) (Borges et al., 2026).

#### 7.2.3 Melatonergic agents in non-surgical populations

The strongest single-trial result comes from a medical rather than a surgical population: among 67 acutely admitted older adults, ramelteon 8 mg nightly for up to 7 days reduced delirium from 32% (11/34) to 3% (1/33), an adjusted odds ratio of 0.07 (95% CI 0.008–0.54) (Hatta et al., 2014). Melatonin reduced delirium in an earlier trial in older medical inpatients (Al-Aama et al., 2011) but not in hospitalized medical patients in a later trial (Jaisw et al., 2018), and it did not reduce the severity of established delirium in a treatment trial (Lange et al., 2024); ramelteon has also been associated with a shorter intensive care stay, which is not direct evidence of delirium prevention (Nishikimi et al., 2018). In the intensive care unit, an earlier meta-analysis found insufficient evidence for routine prophylaxis (Aiello et al., 2023), the DEMEL trial found no benefit of melatonin in mechanically ventilated patients (Mekontso Dessap et al., 2025), and a 2026 dose–response meta-analysis of melatonin or ramelteon reported a modest reduction (risk ratio 0.77, 95% CI 0.62–0.94) based on low-certainty evidence (Chaves-Filho et al., 2026). These results should not be extrapolated directly to surgical patients.

#### 7.2.4 Pooled melatonergic analyses

Meta-analyses combining melatonin and ramelteon remain divergent: an earlier meta-analysis reported a possible preventive effect in older surgical patients (Campbell et al., 2019), and a later synthesis found a lower pooled delirium incidence with stronger subgroup signals for higher-dose melatonin and cardiopulmonary surgery (Shin et al., 2024). Pooled estimates may conceal an agent-specific effect: in a 2026 pairwise meta-analysis of 16 randomized surgical trials, the overall risk ratio of 0.72 was attributable to melatonin trials rather than ramelteon trials (Borges et al., 2026). A Bayesian network meta-analysis in cardiac surgery ranked dexmedetomidine plus melatonin highly, although that estimate depended substantially on indirect, low-certainty comparisons (Queiroz et al., 2026).

#### 7.2.5 Four observations discipline the interpretation

First, dose may be an effect modifier: two meta-analyses reported stronger effects in subgroups receiving at least 5 mg (Borges et al., 2026; Han et al., 2020), and given variable bioavailability and CYP1A2 activity, 3 mg may produce inadequate exposure in some patients (DeMuro et al., 2000; Hartter et al., 2003), although subgroup convergence does not establish a biological threshold. Second, population and intervention class matter: the large effect observed in acutely admitted medical patients (Hatta et al., 2014) has not been reproduced consistently in surgical cohorts, and pooled estimates differ between melatonin and ramelteon (Borges et al., 2026); because postoperative delirium reflects inflammation, pain, opioids, hemodynamic instability, anemia, and baseline vulnerability, a melatonergic intervention would be expected to address only part of the causal burden. Third, timing has rarely been anchored to internal phase, since the major trials did not time dosing to individual dim-light melatonin onset (de Jonghe et al., 2014; Ford et al., 2020; Kinouchi et al., 2023; Oh et al., 2021), and because the melatonin phase–response curve is time dependent, administrative evening dosing is not equivalent to phase-targeted dosing (Burgess et al., 2008). Fourth, postrandomization exclusion of 74 of 452 participants in the largest hip-fracture trial, although clinically explainable, reduced the protection afforded by intention-to-treat allocation (de Jonghe et al., 2014).

Current evidence therefore does not support universal melatonin or ramelteon prophylaxis for postoperative delirium (Borges et al., 2026; Shin et al., 2024), but neither does it exclude benefit from a better-targeted intervention combining phase-aware timing, an appropriate formulation, and verified exposure (Guo et al., 2023; Borges et al., 2026). Overall certainty is low because of inconsistency, imprecision, and indirectness between the regimens tested and the chronobiotic mechanism proposed (Borges et al., 2026; Shin et al., 2024).

Evidence on melatonergic prevention of delayed neurocognitive recovery and postoperative neurocognitive disorder is sparse and is not appraised separately here; the conclusions of this section apply to postoperative delirium only.

### 7.3 Postoperative sleep and rest–activity rhythms

Effects of melatonin on postoperative sleep are modest and inconsistently measured, predominantly by questionnaire rather than by actigraphy or polysomnography, and rarely with rhythm parameters such as mesor, amplitude, or acrophase (Guo et al., 2023; van et al., 2022). Dexmedetomidine has stronger evidence for selected sleep outcomes (Akeju et al., 2018; Wu et al., 2016), yet a human pharmacological study found no measurable correction of nocturnal melatonin despite effects on sleep architecture (Schnider et al., 2025). Sleep architecture and circadian phase are therefore separable perioperative targets (Section 1.2) that require distinct interventions and endpoints.

### 7.4 Pain and opioid consumption

Perioperative analgesic effects of melatonin have been described as inconsistent (Andersen et al., 2014), and a later meta-analysis found that the apparent benefit attenuated when analyses were restricted to higher-quality double-blind placebo-controlled trials (Oh et al., 2020). Given the short half-life of immediate-release melatonin, a single preoperative dose is unlikely to maintain direct receptor occupancy throughout a 24-h pain endpoint, although downstream effects may persist (DeMuro et al., 2000); trial duration and formulation should therefore be matched to the intended analgesic window.

### 7.5 Safety

Melatonin has a generally favorable short-term safety profile in adults: reported adverse effects include nausea, sleepiness, dizziness, and headache, without serious treatment-related events, and dedicated reviews support short-term tolerability (Madsen et al., 2020; Andersen et al., 2016). Three caveats remain. Genotype-dependent glycemic effects related to *MTNR1B* are biologically plausible but have not been measured in perioperative populations (Lyssenko et al., 2009; Garaulet et al., 2015). Residual sleepiness or dizziness may matter in frail patients at risk of delayed mobilization or falls, and existing trials are underpowered for uncommon harms. Finally, in jurisdictions where melatonin is marketed as a supplement rather than a regulated medicine, variability in product content and release characteristics can undermine reproducibility (Erland and Saxena, 2017).

## 8 Clinical implications

Current evidence supports cautious, indication-specific interpretation rather than a universal perioperative melatonin protocol (Borges et al., 2026; Madsen et al., 2020).

### 8.1 What current evidence supports

In adults, melatonin may be considered for preoperative anxiolysis when a non-benzodiazepine option is preferred, provided that product quality, dose, timing, and local prescribing standards are reliable (Madsen et al., 2020). For environmental circadian protection, adequate daytime light, nocturnal darkness and quiet, clustering of overnight care, early mobilization, appropriate analgesia, and structured feeding have a strong mechanistic rationale and low implementation risk, although outcome-specific trial evidence remains incomplete (Wilcox et al., 2024). Dexmedetomidine has evidence for improving selected sleep outcomes (Akeju et al., 2018; Wu et al., 2016) and reduced delirium in some but not all settings (Su et al., 2016; Deiner et al., 2017); these effects should not be interpreted as proof that the melatonin rhythm has been restored. Recording surgery time, drug-administration time, light exposure, and sleep opportunity is a low-cost minimum standard for clinical studies and quality-improvement programs alike.

### 8.2 What is not yet supported

Current evidence does not support routine melatonin or ramelteon prophylaxis for postoperative delirium (Borges et al., 2026; Shin et al., 2024), selection of anesthetic technique solely to preserve circadian rhythm (Guo et al., 2023; Sugimura et al., 2024), or routine rescheduling of cardiac surgery to the afternoon as a cardioprotective strategy (Meewisse et al., 2024; Nemeth et al., 2021; Gotte et al., 2020). Use of *MTNR1B* genotype, chronotype, or dim-light melatonin onset to guide clinical dosing is not justified outside research settings (Murray et al., 2024; Lyssenko et al., 2009; Garaulet et al., 2015), and claims of organ protection based on short-term changes in melatonin concentrations, cytokines, or oxidative-stress biomarkers remain unsubstantiated (Reiter et al., 2016; Tan et al., 2016).

### 8.3 Practical considerations when melatonin is used

Melatonin exposure can be materially altered by formulation, release profile, administration time, fasting status, hepatic function, CYP1A2 inhibitors or inducers, and changes in smoking or caffeine intake (Lane and Moss, 1985; Hartter et al., 2003). In high-risk patients, melatonin should complement rather than replace multicomponent delirium prevention, management of sleep apnea, opioid-sparing analgesia, hemodynamic optimization, and treatment of infection or organ dysfunction (Borges et al., 2026; Shin et al., 2024).

## 9 Toward precision perioperative chronomedicine

### 9.1 Phenotype before treating

A precision approach should characterize chronotype, habitual sleep timing, recent shift work or jet lag, baseline insomnia, obstructive sleep apnea, frailty, cognition, sensory impairment, diabetes, kidney and liver function, β-blocker use, CYP1A2-modifying exposures, and habitual melatonin or receptor-agonist use (Figure 5; Table 6) (Murray et al., 2024; Lane and Moss, 1985; Hartter et al., 2003; Nathan et al., 1997; Karel et al., 2024; Ghotbi et al., 2020). These features may alter endogenous rhythm, treatment exposure, competing risk, or the interpretation of a postoperative melatonin measurement (Guo et al., 2023; Lyssenko et al., 2009).

**FIGURE 5:**
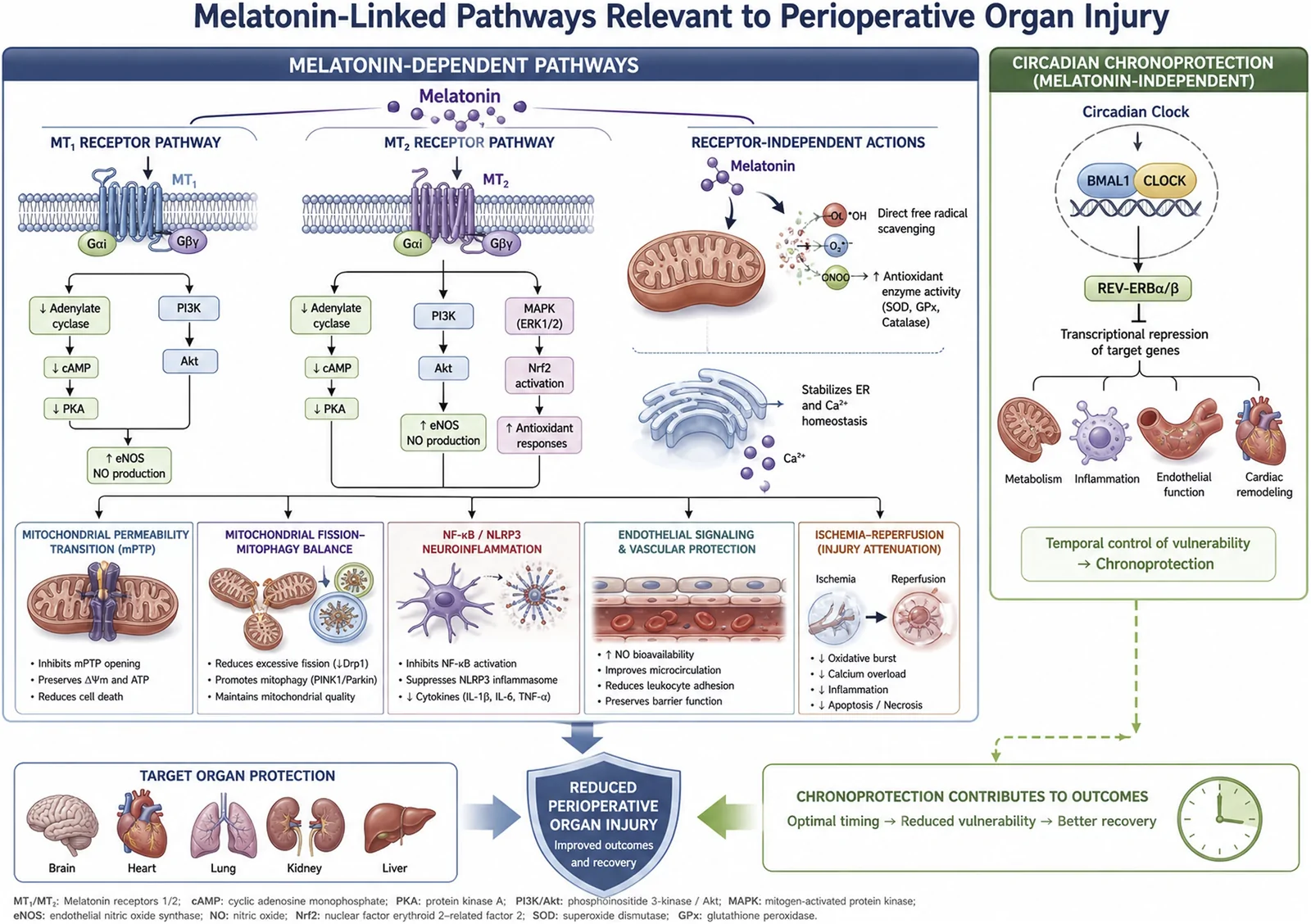
Proposed precision perioperative chronomedicine framework. Research algorithm linking phenotype (chronotype, frailty, cognition, sleep apnea, metabolic disease), internal phase, formulation and exposure, anesthetic and environmental schedule, and outcome-specific endpoints. The framework is hypothesis-generating and has not been validated for routine clinical use.

**TABLE 6:** Proposed precision-perioperative research framework.

Domain	Candidate measure	Measurement	Research use	References
Phase	Dim-light melatonin onset (DLMO)	Serial salivary melatonin sampling under controlled dim light across the expected onset window; validated at-home protocols may reduce burden. Actigraphy and photometry are complementary estimators, not direct DLMO measures	Use DLMO to define internal circadian phase and prospectively test DLMO-relative dosing against fixed clock-time dosing and placebo	DLMO-timed perioperative RCT (Gap 1) Laboratory DLMO method: (St Hilaire and Lockley, 2022) At-home DLMO protocol: (Murray et al., 2024) Experimental wearable estimator under circadian alignment: (Brown et al., 2021)
Exposure	Plasma melatonin C_max_/AUC; formulation	Sparse population-PK sampling with exact dose, formulation, administration time, fed state, and relevant co-medications	Relate exposure to the intended mechanism; do not assume immediate- and prolonged-release products provide equivalent exposure profiles	Population-PK and exposure-response substudy (Gap 2) Oral bioavailability study: (DeMuro et al., 2000) Systematic human PK review: (Harpsoe et al., 2015)
Genotype	*MTNR1B* rs10830963	Preoperative genotyping or existing array data	Use stratified randomization or a prespecified genotype-by-treatment analysis; include perioperative glucose monitoring as a safety endpoint	Genotype-stratified RCT (Gap 3) Genetic association: (Lyssenko et al., 2009) Human islet/signaling study: (Tuomi et al., 2016) Timed melatonin-glucose interaction: (Garaulet et al., 2015)
Metabolism	CYP1A2-modifying exposures (smoking, caffeine, fluvoxamine or other inhibitors); exploratory CYP1A2 genotype	Structured smoking, caffeine, and medication history; optional genotype and sparse melatonin PK where feasible	Treat these factors as exposure covariates. Genotype-guided or exposure-guided dosing requires prospective validation	PK substudy; no validated perioperative dosing algorithm Caffeine interaction: (Hartter et al., 2003) Fluvoxamine interaction: (Hartter et al., 2000)
Chronotype	MEQ or MCTQ (MSF_sc_)	Brief preoperative questionnaire; wearable light/activity data may be collected as exploratory measures	Report chronotype as a covariate and prespecify a chronotype-by-time-of-surgery interaction	Prospective perioperative interaction analysis Ultra-short MCTQ validation: (Ghotbi et al., 2020) Experimental wearable phase estimation: (Brown et al., 2021)
Anesthetic technique	Technique and agent class	Record neuraxial/general technique, volatile/IV agent, dose, timing, depth, and adjuncts	Compare techniques or agents only with a prespecified circadian endpoint, such as melatonin phase, amplitude, DLMO, or a validated proxy	Mechanistic RCT or cohort with circadian endpoints General vs. subarachnoid anesthesia and melatonin rhythm: (Song et al., 2021) Direct volatile-vs-IV agent comparisons with validated circadian endpoints remain a gap
Timing of surgery	Early vs. late start time	Record actual incision or bypass time and, when feasible, internal circadian phase rather than clock-time categories alone	Treat start time as an exposure. Current evidence does not justify routine clinical chronoscheduling	Adequately powered multicenter RCTs with patient-important outcomes Updated Cochrane review (2026): (Liu et al., 2026)
Outcome-matched agent	Target-defined intervention	Prespecify the primary mechanism and outcome	Anxiolysis: melatonin. Sleep architecture: dexmedetomidine. Re-entrainment: phase-timed melatonergic intervention (unproven perioperatively). Cardioprotection: REV-ERB-directed strategies remain investigational	Indication-specific trials Anxiolysis systematic review: (Madsen et al., 2020) Dexmedetomidine sleep physiology/RCTs: (Akeju et al., 2018; Wu et al., 2016) Cardiac-surgery/REV-ERB translational study: (Montaigne et al., 2018) None validates routine precision selection
Environment	Light exposure and nocturnal interruptions	Wearable or bedside light dosimetry; audit nighttime interruptions and care clustering	Test multicomponent circadian-hygiene bundles, including daytime light, nighttime darkness/quiet, and clustered care	Cluster RCT with fidelity measurement and circadian plus clinical outcomes ICU sleep/circadian-delirium scoping review: (Wilcox et al., 2024) ICU environmental-light study: (Fan et al., 2017)

DLMO, dim-light melatonin onset; IV, intravenous; MCTQ, Munich ChronoType Questionnaire; MEQ, Morningness-Eveningness Questionnaire; PK, pharmacokinetics. All proposed actions are research hypotheses and are not validated for routine care.

### 9.2 Define the therapeutic objective

Anxiolysis, sleep promotion, circadian-phase correction, analgesic adjunctive effect, and higher-exposure experimental cytoprotection are related but non-equivalent objectives (Reiter et al., 2016; Tan et al., 2016). Because immediate-release and prolonged-release preparations generate different exposure profiles, and because clock time is not equivalent to internal circadian phase, dosing research should be designed around the intended biological target rather than a single empirical bedtime schedule (DeMuro et al., 2000). Re-entrainment trials should also specify the agent tested, because the pharmacokinetic profile of ramelteon and the absence of demonstrated receptor-independent actions make it a different intervention from high-dose melatonin (Table 2).

### 9.3 Align drug, surgery, and environment

The research intervention should combine phase-aware drug administration with standardized light, feeding, mobilization, temperature, and sleep-protection protocols, treating anesthetic choice as one component of a multimodal schedule rather than as a stand-alone circadian therapy (Guo et al., 2023; van et al., 2022). Environmental care should itself be measured, using light dosimetry, nursing-interruption logs, actigraphy, and analgesic exposure (Guo et al., 2023; van et al., 2022).

### 9.4 Measure exposure and mechanism

Future trials should include sparse pharmacokinetic sampling, prespecified formulation and meal state, dim-light melatonin onset or a validated complementary phase measure, actigraphy, standardized delirium assessment, and mechanistic biomarkers (Murray et al., 2024; DeMuro et al., 2000; St Hilaire and Lockley, 2022). *MTNR1B* and CYP1A2 analyses should initially be exploratory and accompanied by glycemic monitoring rather than used as unvalidated treatment rules.

### 9.5 Match trial design to the outcome

An anxiolysis trial requires minutes-to-hours exposure and patient-reported anxiety; a sleep trial requires overnight architecture and rhythm measures; a delirium trial requires repeated validated assessment over several days; and an organ-protection trial requires adequately powered clinical endpoints with prespecified mediation analyses (Guo et al., 2023; Shin et al., 2024). Mixing these objectives obscures both efficacy and failure (Section 1.2).

## 10 Research priorities

Several gaps are tractable with existing methods. No adequately powered perioperative trial has compared dosing anchored to individual dim-light melatonin onset with fixed clock-time dosing and placebo; such a trial should combine prolonged-release pharmacology, actigraphic phase outcomes, validated delirium assessment, and prespecified environmental control (Guo et al., 2023; Borges et al., 2026). Because perioperative trials rarely measure plasma melatonin, population pharmacokinetic modeling with sparse sampling is needed to distinguish a mechanism-negative from an exposure-negative intervention (DeMuro et al., 2000; Hartter et al., 2003). *MTNR1B* rs10830963 and CYP1A2-related variability are plausible effect modifiers that genotype-stratified analyses with continuous or frequent glucose monitoring should test rather than assume (Lyssenko et al., 2009; Garaulet et al., 2015). Rodent data showing phase-dependent effects of sevoflurane and desflurane (Kadota et al., 2012; Imai et al., 2020) could be extended by carefully designed volunteer or low-risk procedural studies determining whether brief anesthetic exposure shifts human dim-light melatonin onset or rest–activity phase.

Comparative and mechanistic work is also lacking. No definitive trial has compared sevoflurane, propofol, and newer agents with circadian phase as a primary endpoint (Guo et al., 2023; Sugimura et al., 2024), and direct circadian and melatonergic data for remimazolam and other newer hypnotics are insufficient, so clock-gene and phase outcomes should be incorporated early in translational development (Guo et al., 2023; Poulsen et al., 2018). Because organ clocks may recover at different rates after anesthesia (Yamaguchi et al., 2022), multi-marker phase estimation could establish whether internal desynchrony rather than uniform phase delay is the clinically relevant pathology. MT_1_ and MT_2_ structural biology creates opportunities for subtype-selective or biased ligands (Stauch et al., 2019; Stein et al., 2020), and REV-ERB-directed strategies may offer chronoprotection without surgical rescheduling, although the current evidence is preclinical and no perioperative ligand has established clinical efficacy (Montaigne et al., 2018). Finally, a core outcome set should specify reporting of surgery and dosing time, light exposure, phase assessment, actigraphy, sleep architecture, delirium instrument and assessment frequency, analgesic exposure, and longer-term neurocognitive outcomes (Guo et al., 2023; Shin et al., 2024).

## 11 Limitations

This review is structured and critical but not systematic; although the search strategy and prioritization principles are reported (Section 1.1), study selection and emphasis reflect author judgment, and neither risk of bias nor publication bias was formally assessed (Guo et al., 2023; Poulsen et al., 2018). The mechanistic evidence is predominantly preclinical, and species differences in SCN organization, melatonin physiology, anesthetic potency, and exposure limit direct translation (Hastings et al., 2018; Poulsen et al., 2018). In clinical studies, anesthesia, surgical injury, opioids, fasting, inflammation, environmental light, and illness severity are difficult to disentangle (Olofsson et al., 2004; Mundigler et al., 2002), so observational associations cannot determine whether melatonin suppression is a cause, mediator, marker, or consequence. Heterogeneity in formulation, dose, timing, surgery, anesthetic regimen, outcome measurement, and follow-up limits pooled inference (Borges et al., 2026; Shin et al., 2024). The precision framework proposed here is accordingly hypothesis-generating: phase-anchored dosing, exposure-guided formulation selection, and genotype-stratified treatment have not been prospectively validated (DeMuro et al., 2000).

## 12 Conclusion

The circadian–melatonin axis plausibly links anesthesia and surgical stress to postoperative sleep–wake disturbance, neuroendocrine and inflammatory responses, metabolism, and mitochondrial function (Guo et al., 2023; van et al., 2022; Song et al., 2021). In humans, these links rest mainly on observational associations. Rodent studies suggest agent-specific clock effects: sevoflurane was associated with suppressed suprachiasmatic *Per2* expression through reduced CLOCK occupancy and histone H4 acetylation, and propofol with altered *Per*/*Cry* expression and CaMK–CREB, AMPK, SIRT1, and GSK-3β signaling (Mori et al., 2014; Yin et al., 2022); these findings await independent replication and human confirmation. In humans, the independent contribution of anesthesia remains difficult to separate from surgical trauma, analgesia, fasting, illness severity, and environmental desynchronization. Melatonin acts through MT_1_ and MT_2_ receptors and may also exert receptor-independent mitochondrial and antioxidant effects at high experimental exposures, which have not been demonstrated clinically, but low-dose chronobiotic treatment should not be equated with the higher exposures used in experimental cytoprotection, particularly given the variability of oral bioavailability, formulation, and CYP1A2-dependent clearance (Dubocovich et al., 2010; Reiter et al., 2016; Su et al., 2022; Yiang et al., 2023).

Adult preoperative anxiolysis is the most consistently supported perioperative indication (Madsen et al., 2020), whereas evidence for postoperative sleep improvement, analgesia, and delirium prevention remains heterogeneous (Shin et al., 2024; Tsukinaga et al., 2023; Oh et al., 2020). A recent meta-analysis suggested a possible melatonin-specific delirium benefit, particularly at doses of 5 mg or more, without a consistent ramelteon effect, but certainty was low and the finding does not support universal prophylaxis (Borges et al., 2026). Progress will depend on trials that measure internal phase, achieved exposure, chronotype, perioperative timing, and environmental zeitgebers; dim-light melatonin onset is a validated phase marker, but phase-anchored treatment remains investigational (Murray et al., 2024; Harpsoe et al., 2015).

## Glossary

AANAT: arylalkylamine N-acetyltransferase
AMPK: AMP-activated protein kinase
ASMT: acetylserotonin O-methyltransferase
ASA: American Society of Anesthesiologists
AUC: area under the curve
BDNF: brain-derived neurotrophic factor
BMAL1 (ARNTL): brain and muscle ARNT-like 1
CaMK: calcium/calmodulin-dependent protein kinase
cGAS: cyclic GMP-AMP synthase
CI: confidence interval
CLOCK: circadian locomotor output cycles kaput
CREB: cAMP response element-binding protein
CRY: cryptochrome
CYP1A2: cytochrome P450 1A2
DLMO: dim-light melatonin onset
dNCR: delayed neurocognitive recovery
Drp1: dynamin-related protein 1
GABA: gamma-aminobutyric acid
GIRK: G-protein-gated inwardly rectifying potassium channel
GPCR: G-protein-coupled receptor
GSK-3β: glycogen synthase kinase-3β
HPA: hypothalamic-pituitary-adrenal
ICU: intensive care unit
IL: interleukin
I/R: ischemia–reperfusion
MAPK: mitogen-activated protein kinase
MCTQ: Munich ChronoType Questionnaire
MEQ: Morningness–Eveningness Questionnaire
mPTP: mitochondrial permeability transition pore
**MT**_**1**_ **/MT**_**2**_: melatonin receptor 1/2
MTNR1A/MTNR1B: melatonin receptor 1A/1B gene
mTOR: mechanistic target of rapamycin
**NAD** ^**+**^: nicotinamide adenine dinucleotide
NF-κB: nuclear factor kappa B
NLRP3: NOD-like receptor family pyrin domain containing 3
NMDA: N-methyl-D-aspartate
NREM: non-rapid eye movement
OSA: obstructive sleep apnea
PACAP: pituitary adenylate cyclase-activating polypeptide
PER: period
PGAM5: phosphoglycerate mutase family member 5
PKA: protein kinase A
PLCβ: phospholipase Cβ
PND: perioperative neurocognitive disorders
POCD: postoperative cognitive dysfunction
POD: postoperative delirium
QR2: quinone reductase 2
REM: rapid eye movement
REV-ERBα (NR1D1): nuclear receptor subfamily 1 group D member 1
RORα: retinoic acid receptor-related orphan receptor α
RORE: ROR response element
ROS: reactive oxygen species
SCN: suprachiasmatic nucleus
SIRT1: sirtuin 1
STING: stimulator of interferon genes
TNF-α: tumor necrosis factor α
VDAC1: voltage-dependent anion channel 1
VIP: vasoactive intestinal peptide
